# Supplementary figures and images for: CK-666 and CK-869 differentially inhibit Arp2/3 iso-complexes
Source: EMBO Rep. 2024 Jul 15;25(8):7. doi: 10.1038/s44319-024-00201-x (PMC11316031; doi:10.1038/s44319-024-00201-x)

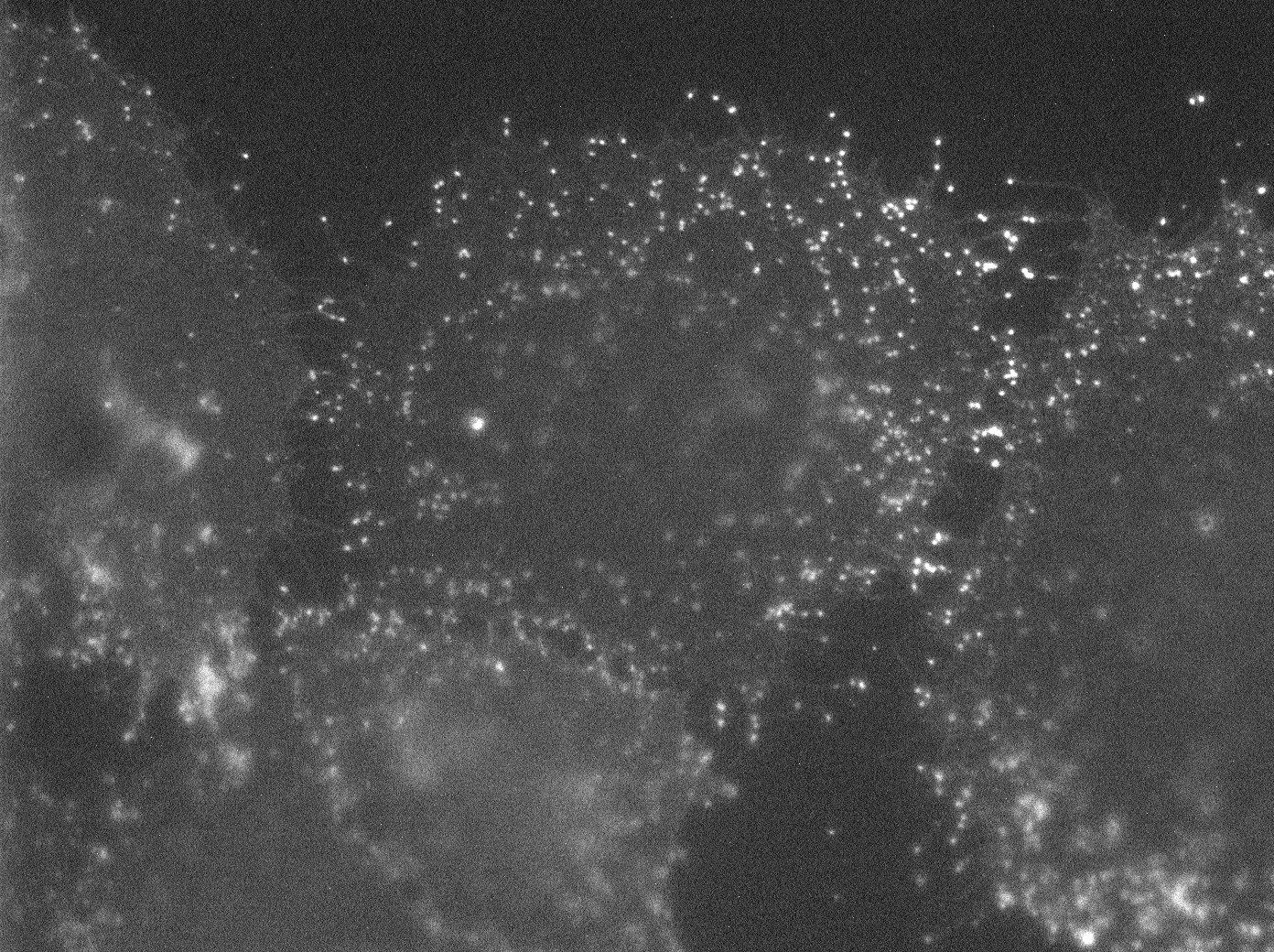

Supplement: Supplementary file 3 — Source data Fig. 1 [file 44319_2024_201_MOESM3_ESM.zip › Figure 1/1A/20210423_HeLa_WR_exB5-647_DMSO.TIF]

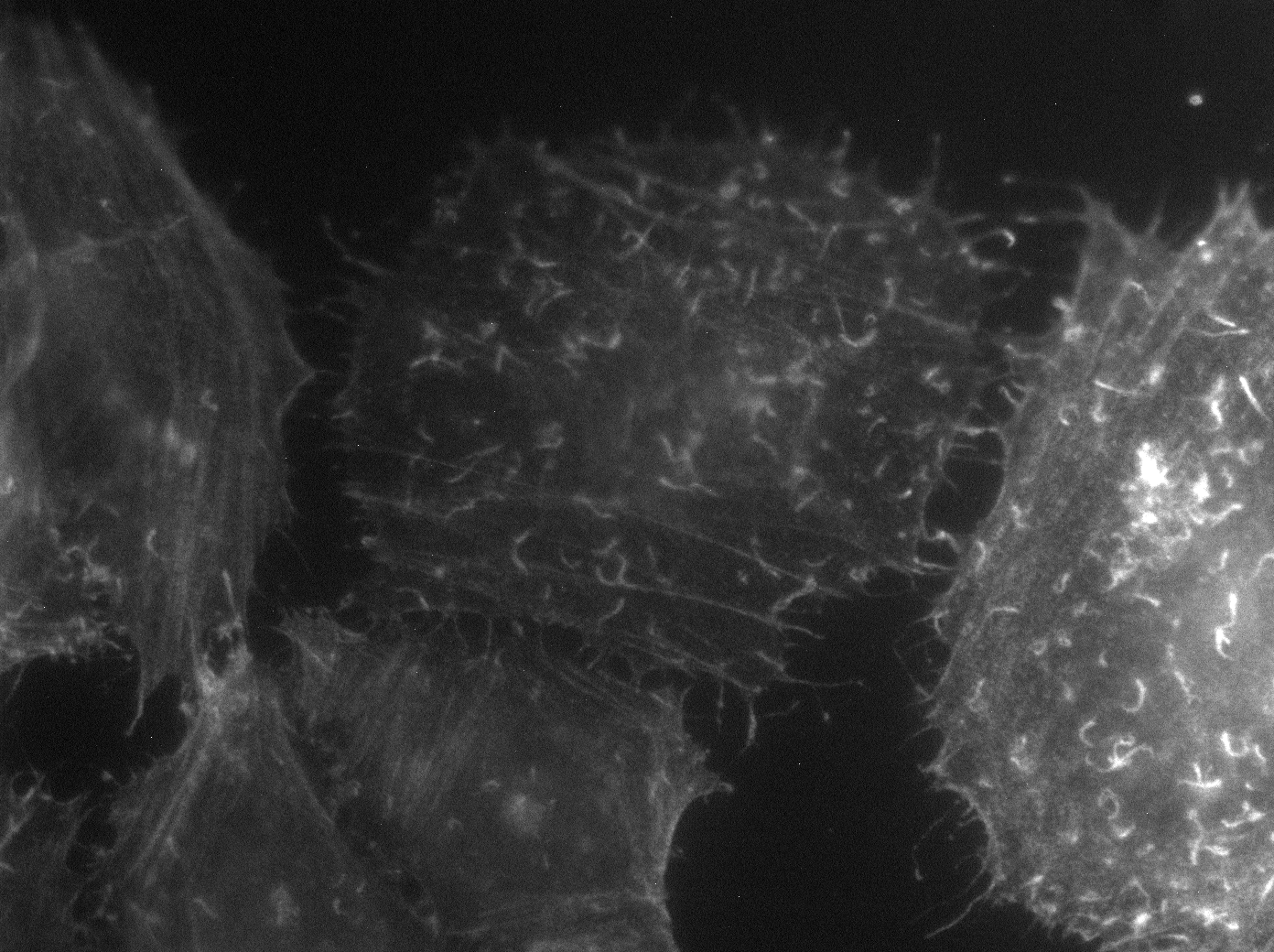

Supplement: Supplementary file 3 — Source data Fig. 1 [file 44319_2024_201_MOESM3_ESM.zip › Figure 1/1A/20210423_HeLa_WR_Phalloidin_DMSO.TIF]

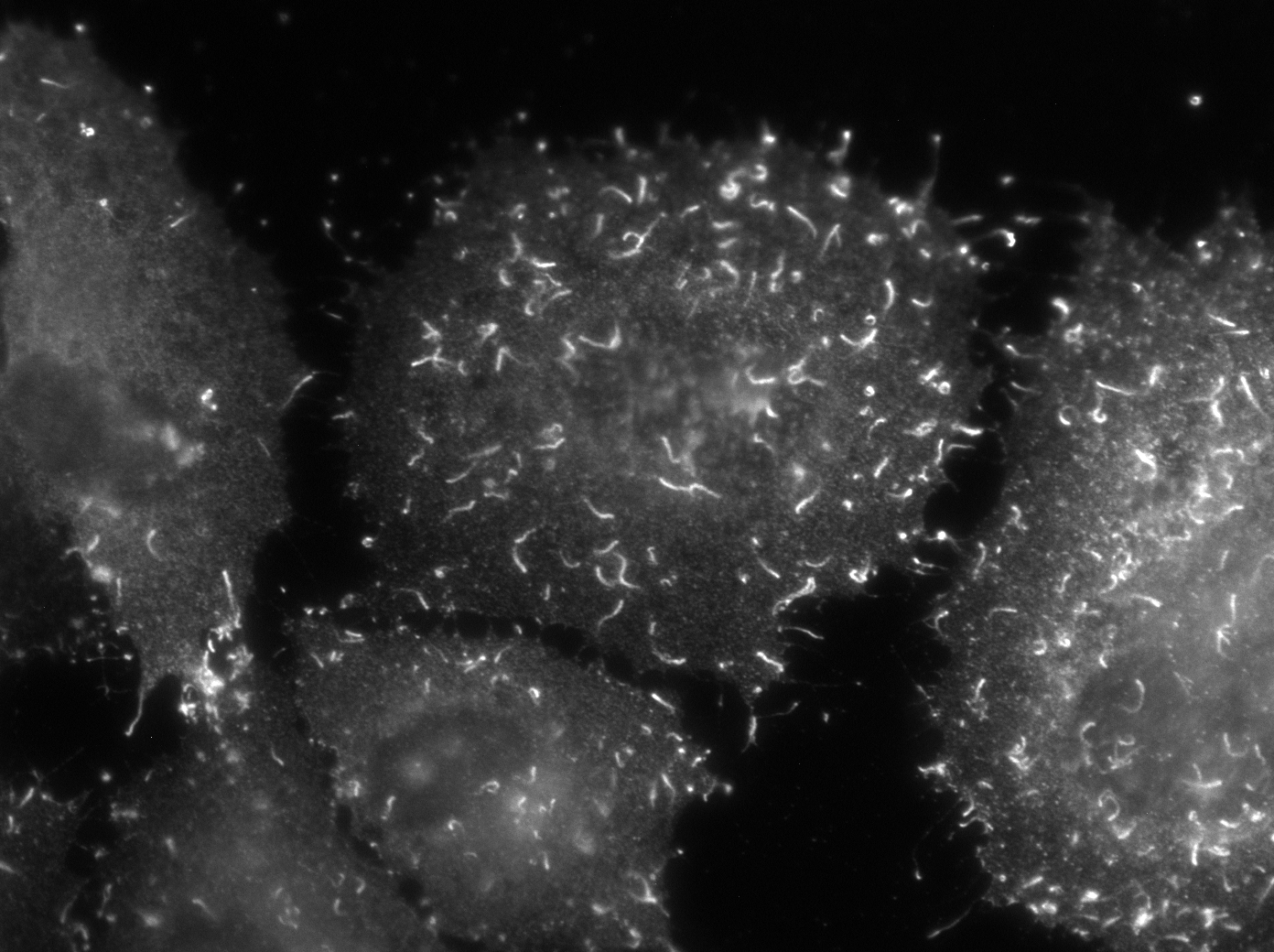

Supplement: Supplementary file 3 — Source data Fig. 1 [file 44319_2024_201_MOESM3_ESM.zip › Figure 1/1A/20210423_HeLa_WR_cortactin_DMSO.TIF]

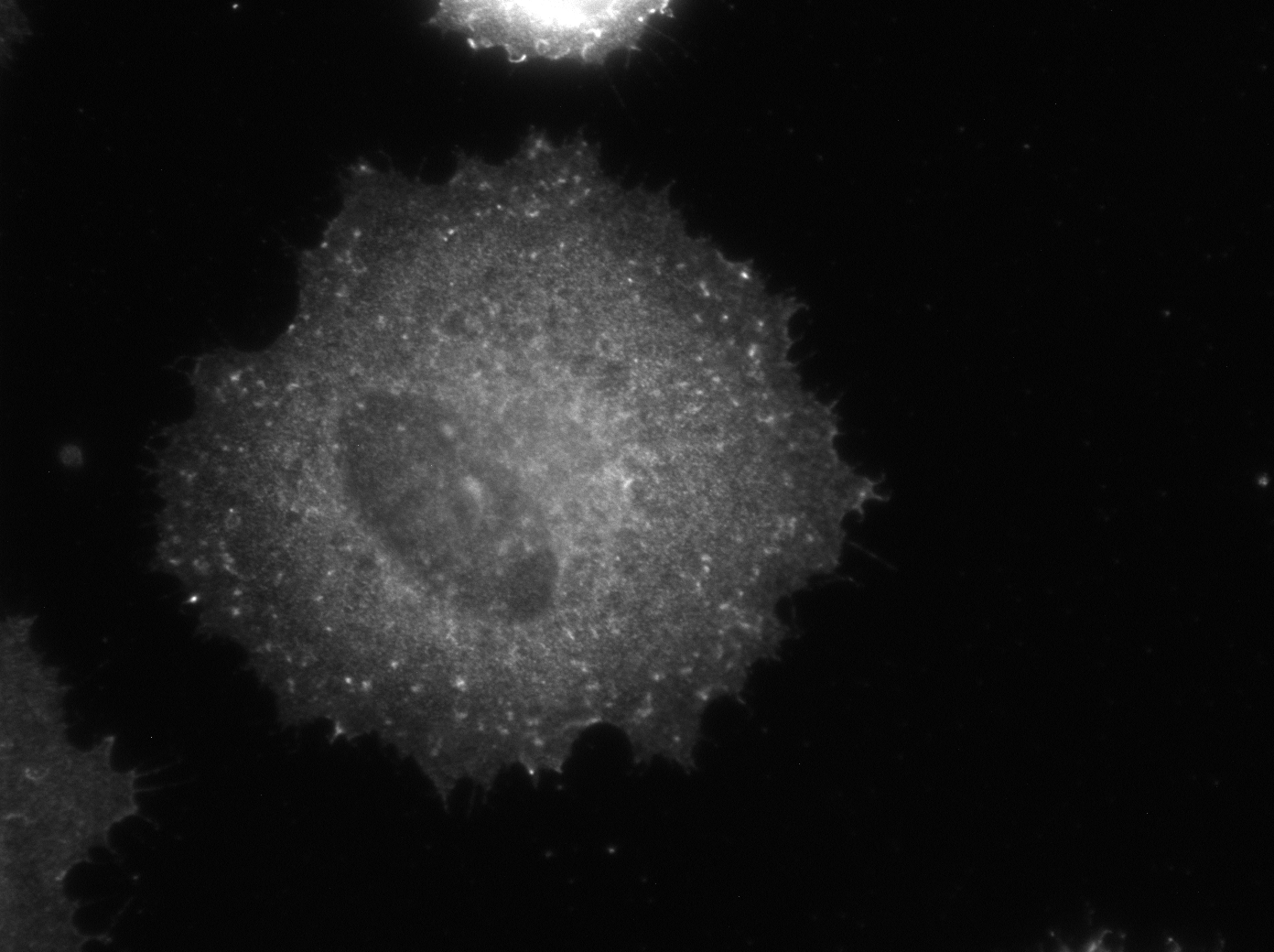

Supplement: Supplementary file 3 — Source data Fig. 1 [file 44319_2024_201_MOESM3_ESM.zip › Figure 1/1B/20210423_HeLa_WR_cortactin ab-488_CK666_50uM.TIF]

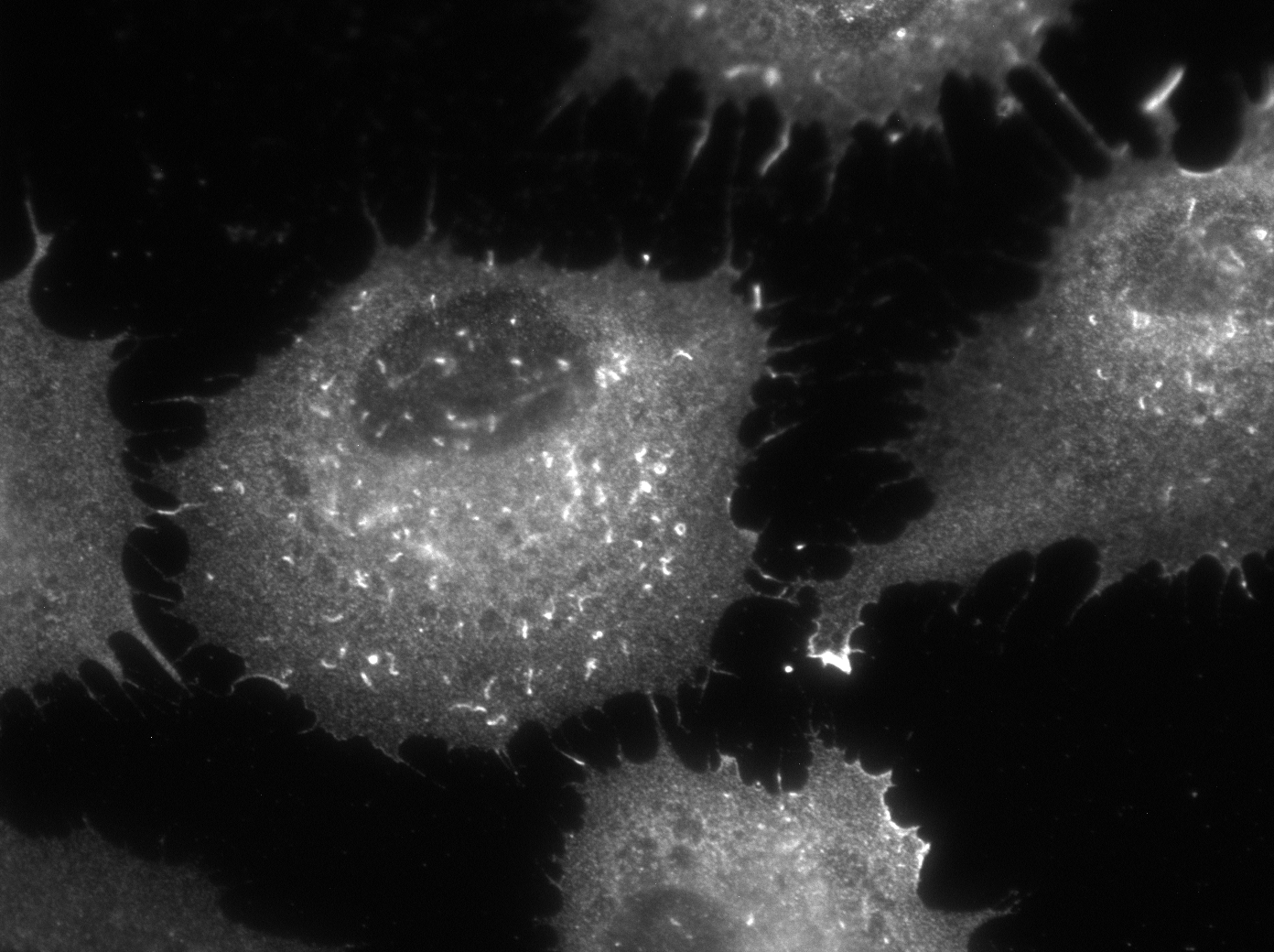

Supplement: Supplementary file 3 — Source data Fig. 1 [file 44319_2024_201_MOESM3_ESM.zip › Figure 1/1B/20210423_HeLa_WR_cortactin ab-488_CK869_50uM.TIF]

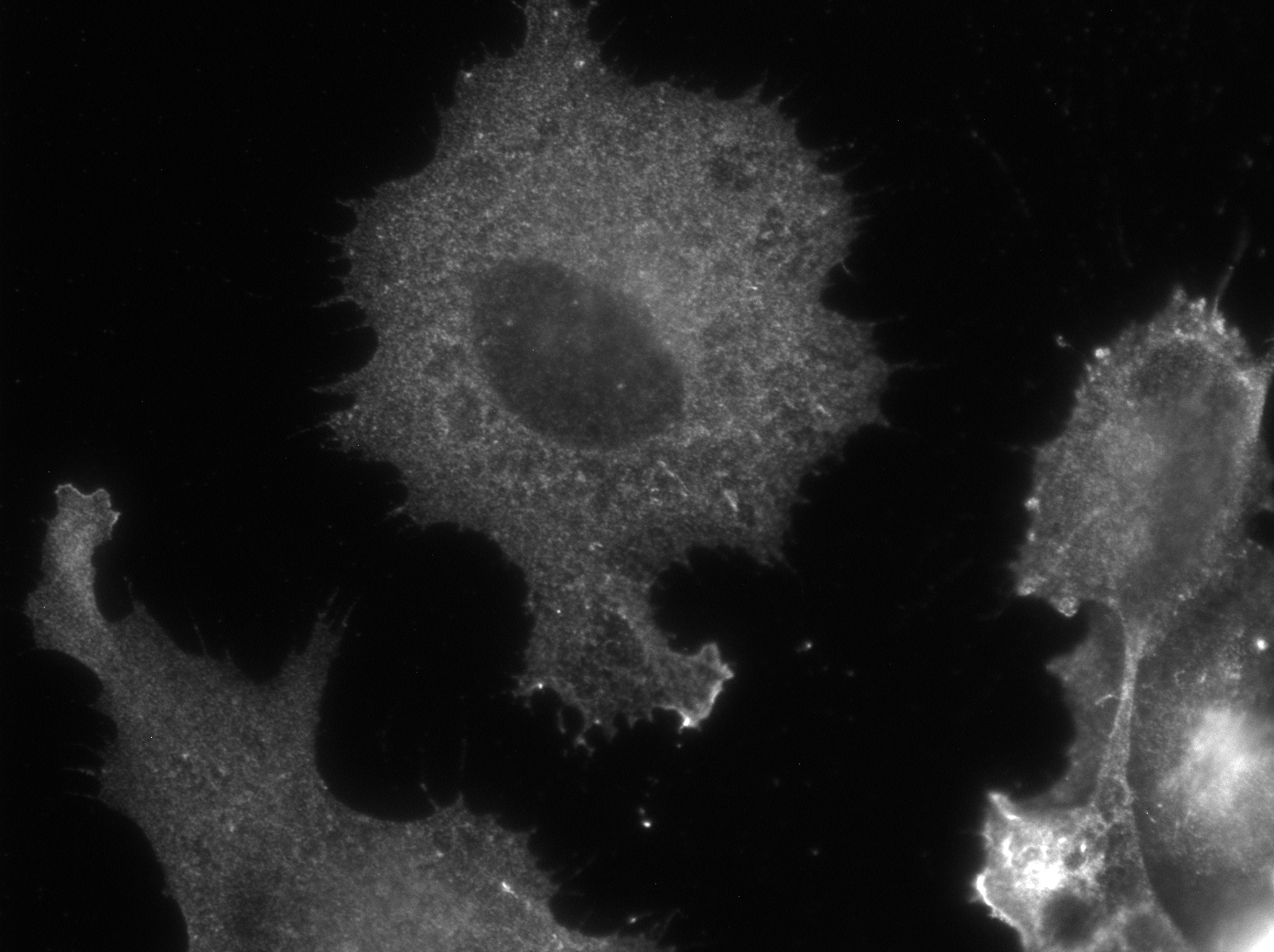

Supplement: Supplementary file 3 — Source data Fig. 1 [file 44319_2024_201_MOESM3_ESM.zip › Figure 1/1B/20210423_HeLa_WR_cortactin ab-488_CK869_300uM.TIF]

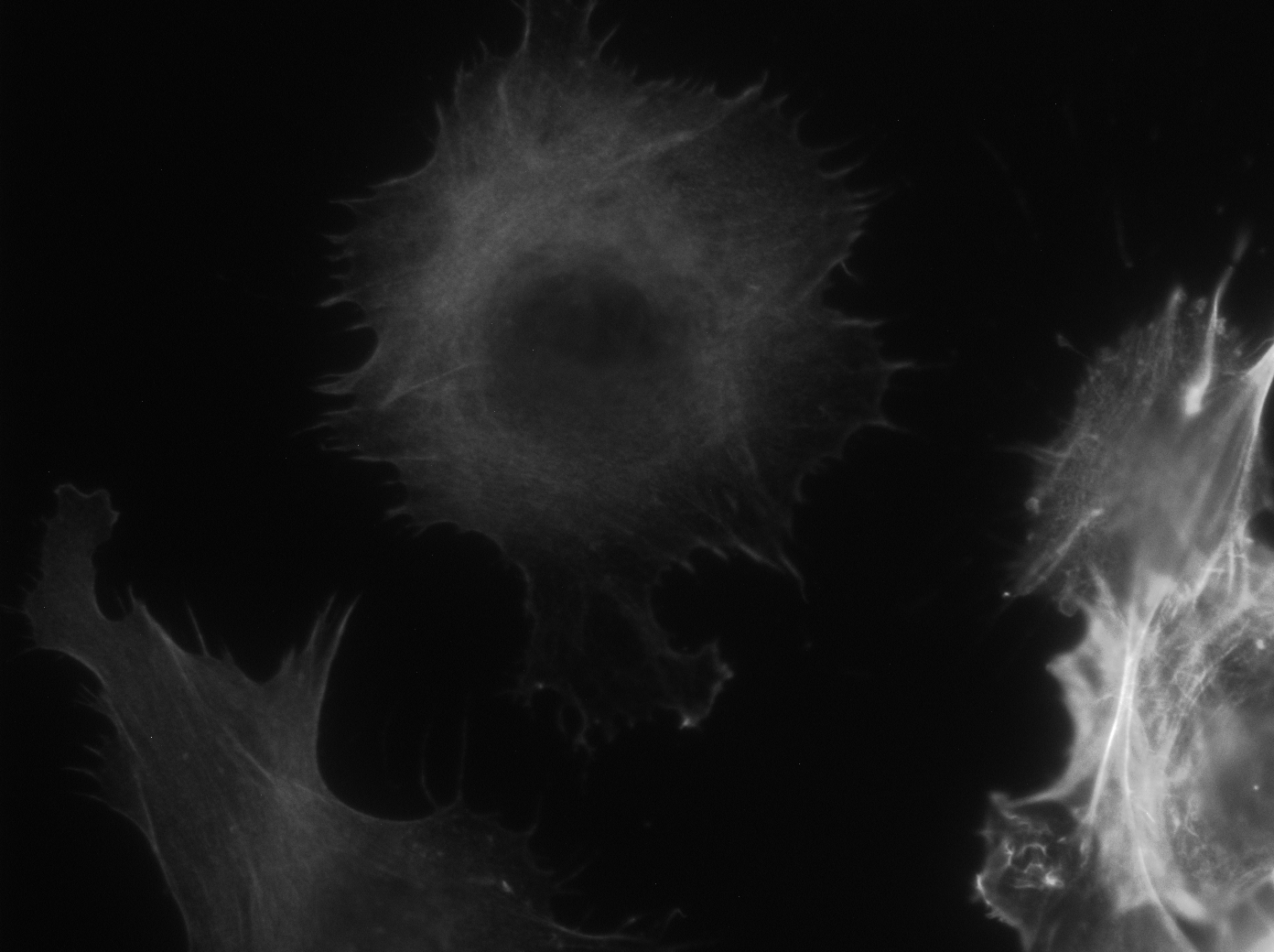

Supplement: Supplementary file 3 — Source data Fig. 1 [file 44319_2024_201_MOESM3_ESM.zip › Figure 1/1B/20210423_HeLa_WR_exB5-647_CK869_300uM.TIF]

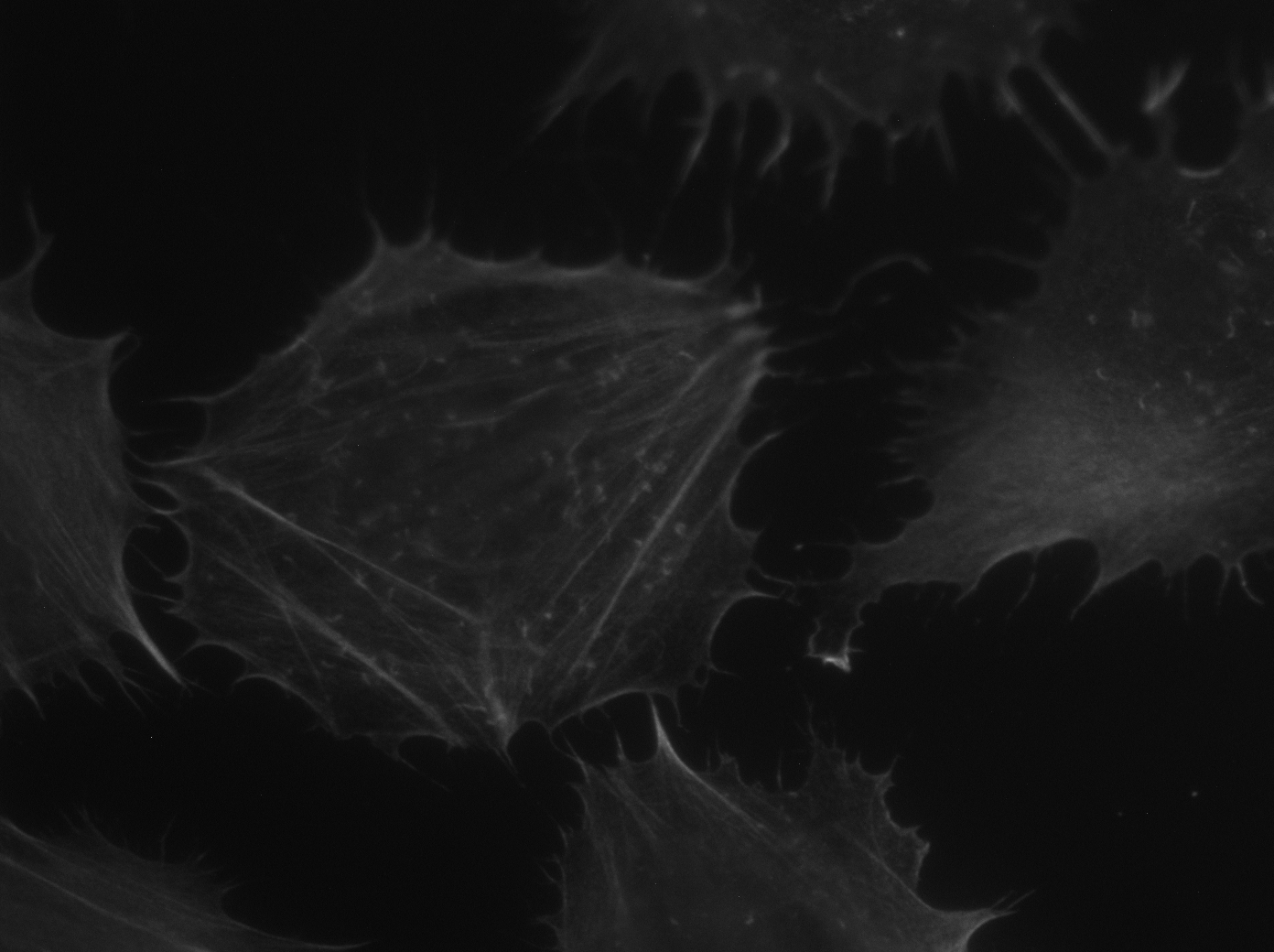

Supplement: Supplementary file 3 — Source data Fig. 1 [file 44319_2024_201_MOESM3_ESM.zip › Figure 1/1B/20210423_HeLa_WR_exB5-647_CK869_50uM.TIF]

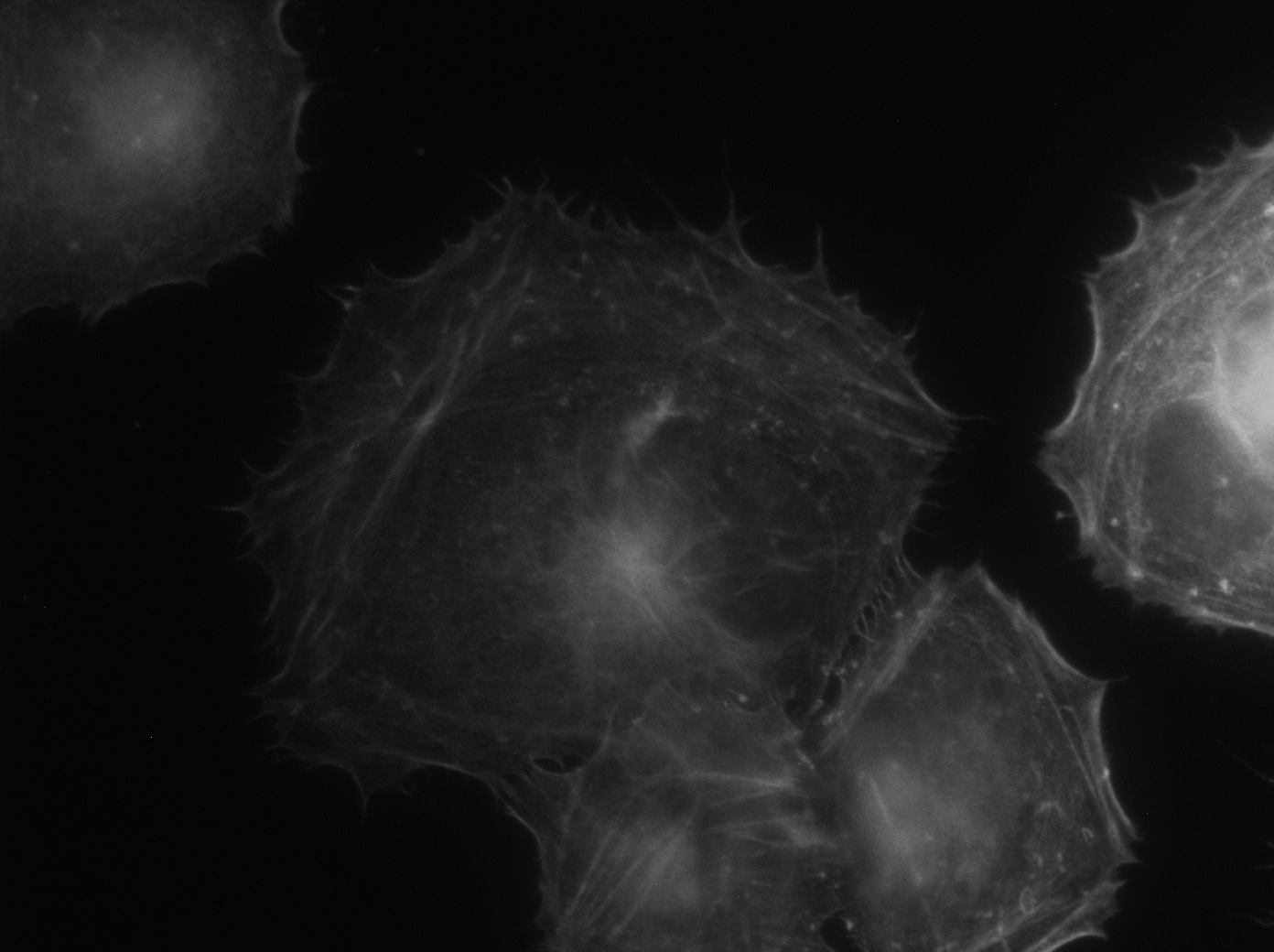

Supplement: Supplementary file 3 — Source data Fig. 1 [file 44319_2024_201_MOESM3_ESM.zip › Figure 1/1B/20210423_HeLa_WR_exB5-647_CK666_100uM.TIF]

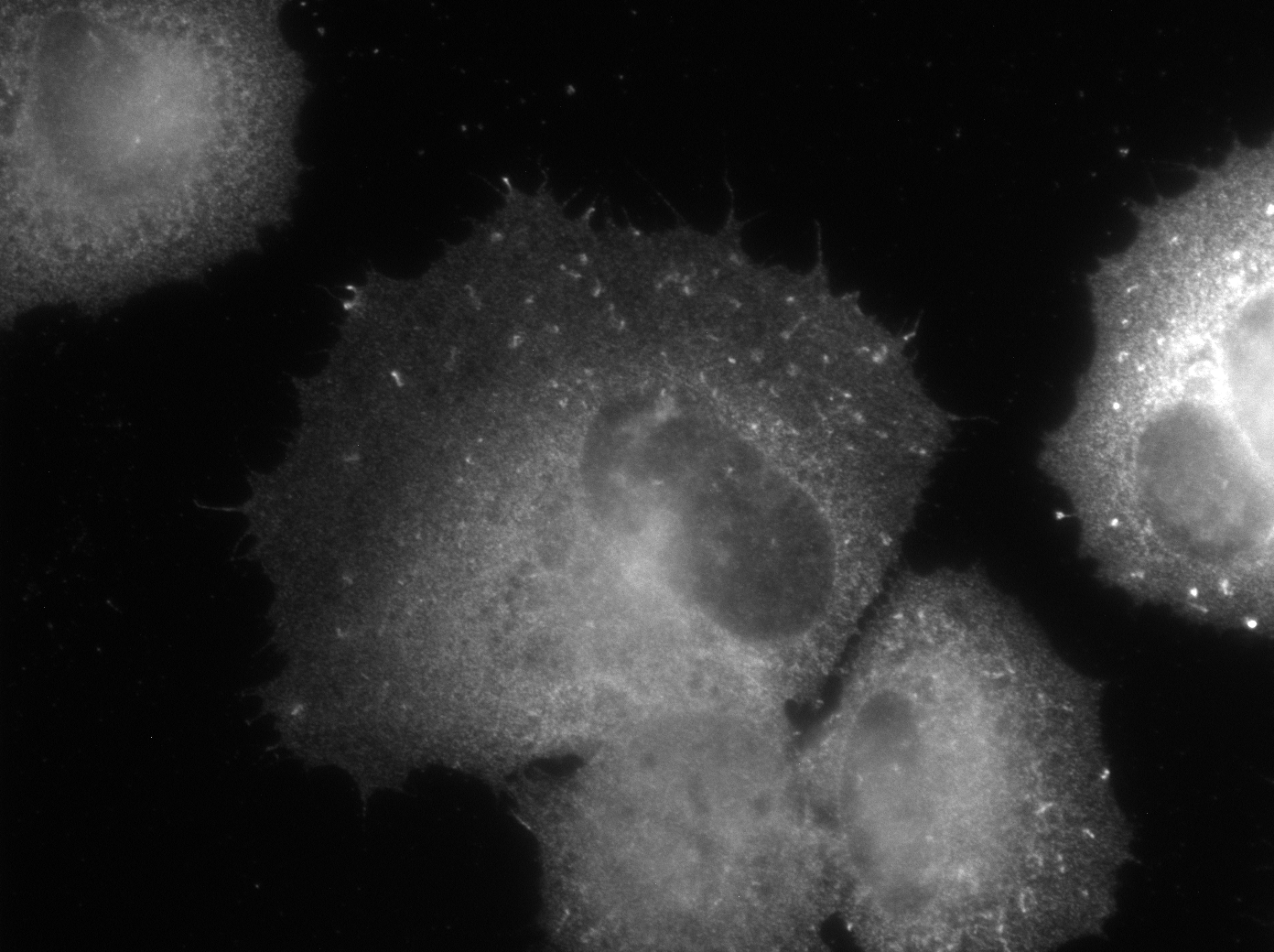

Supplement: Supplementary file 3 — Source data Fig. 1 [file 44319_2024_201_MOESM3_ESM.zip › Figure 1/1B/20210423_HeLa_WR_cortactin ab-488_CK666_100uM.TIF]

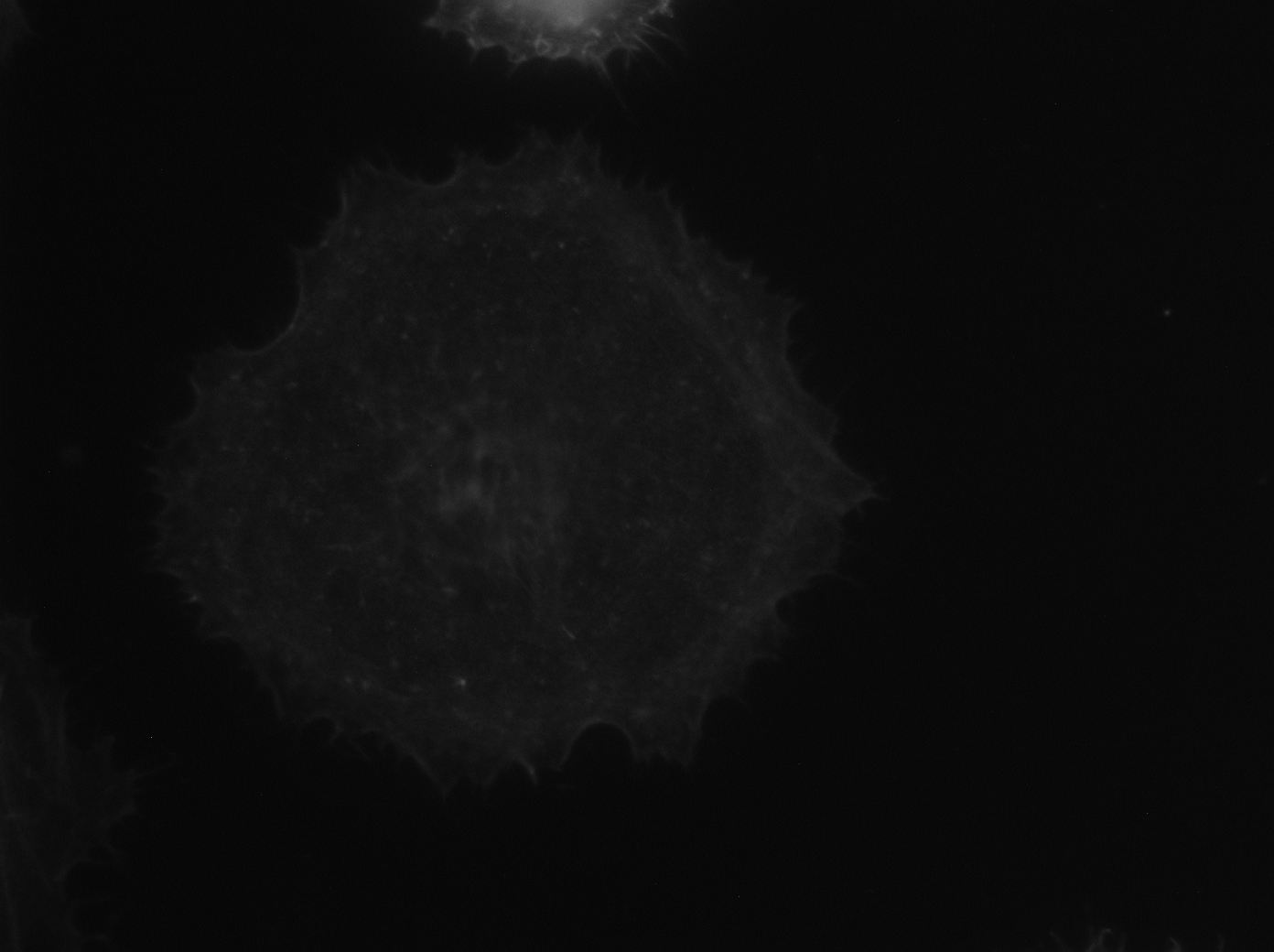

Supplement: Supplementary file 3 — Source data Fig. 1 [file 44319_2024_201_MOESM3_ESM.zip › Figure 1/1B/20210423_HeLa_WR_exB5-647_CK666_50uM.TIF]

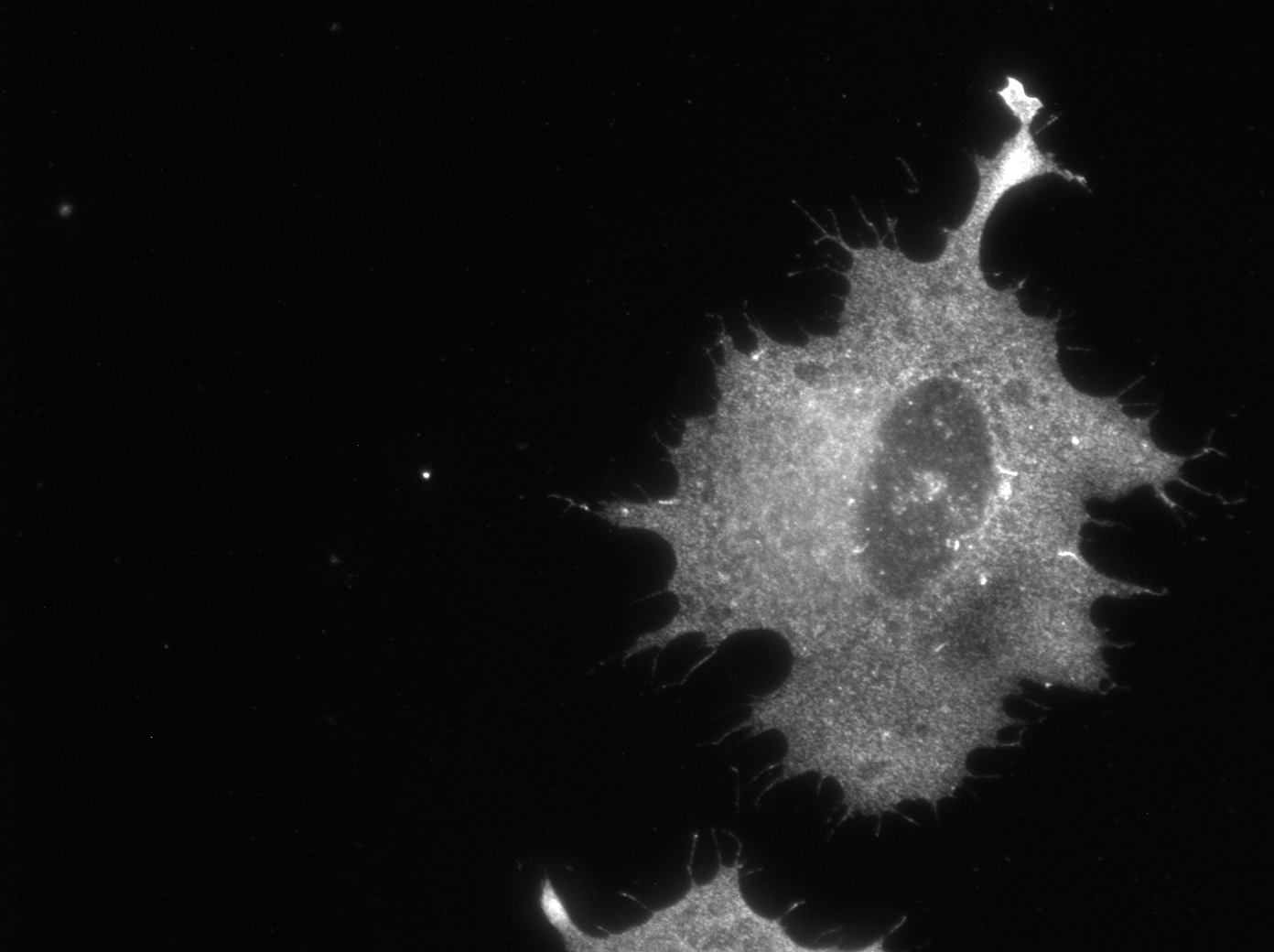

Supplement: Supplementary file 3 — Source data Fig. 1 [file 44319_2024_201_MOESM3_ESM.zip › Figure 1/1B/20210423_HeLa_WR_cortactin ab-488_CK869_200uM.TIF]

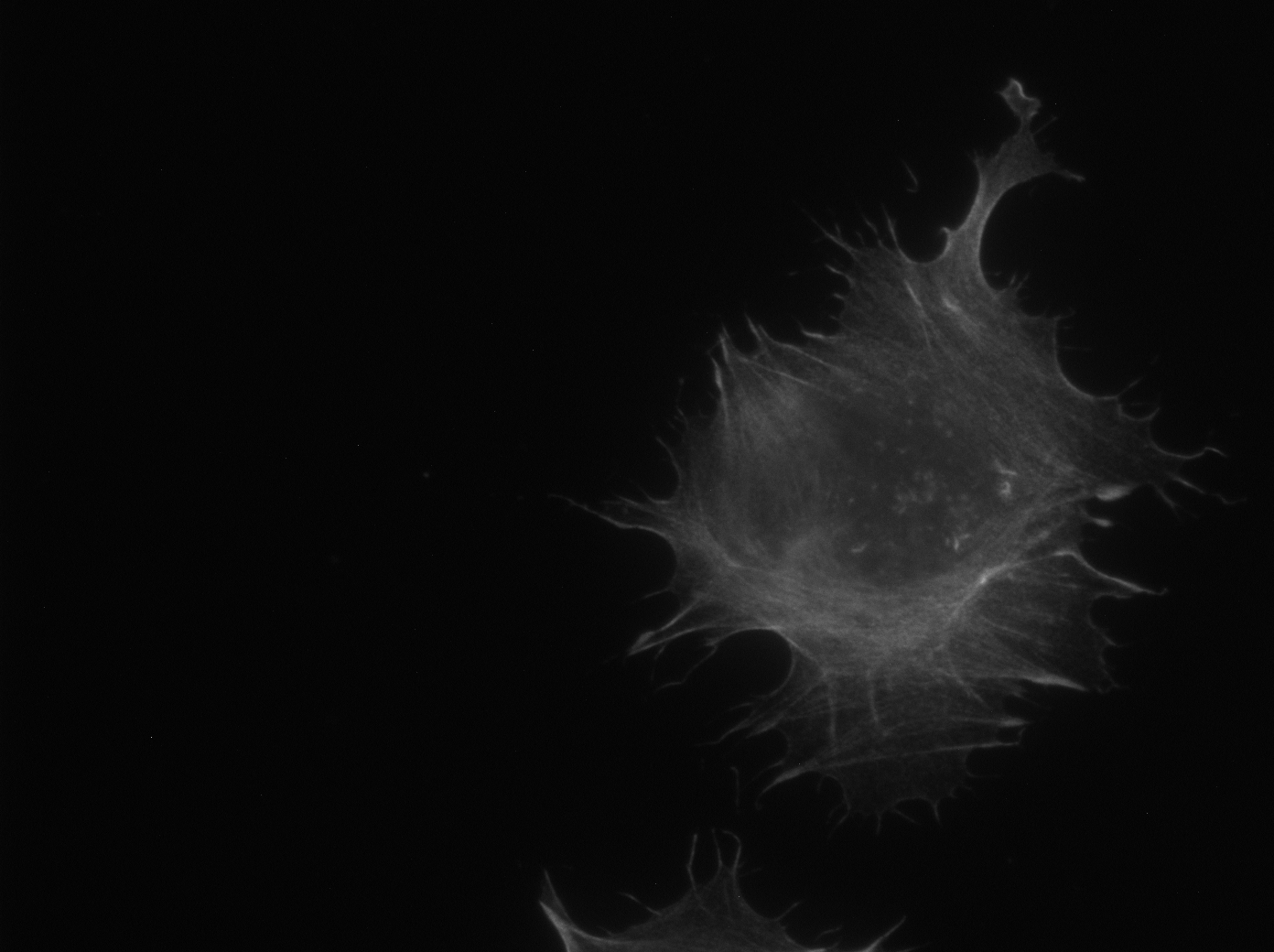

Supplement: Supplementary file 3 — Source data Fig. 1 [file 44319_2024_201_MOESM3_ESM.zip › Figure 1/1B/20210423_HeLa_WR_exB5-647_CK869_200uM.TIF]

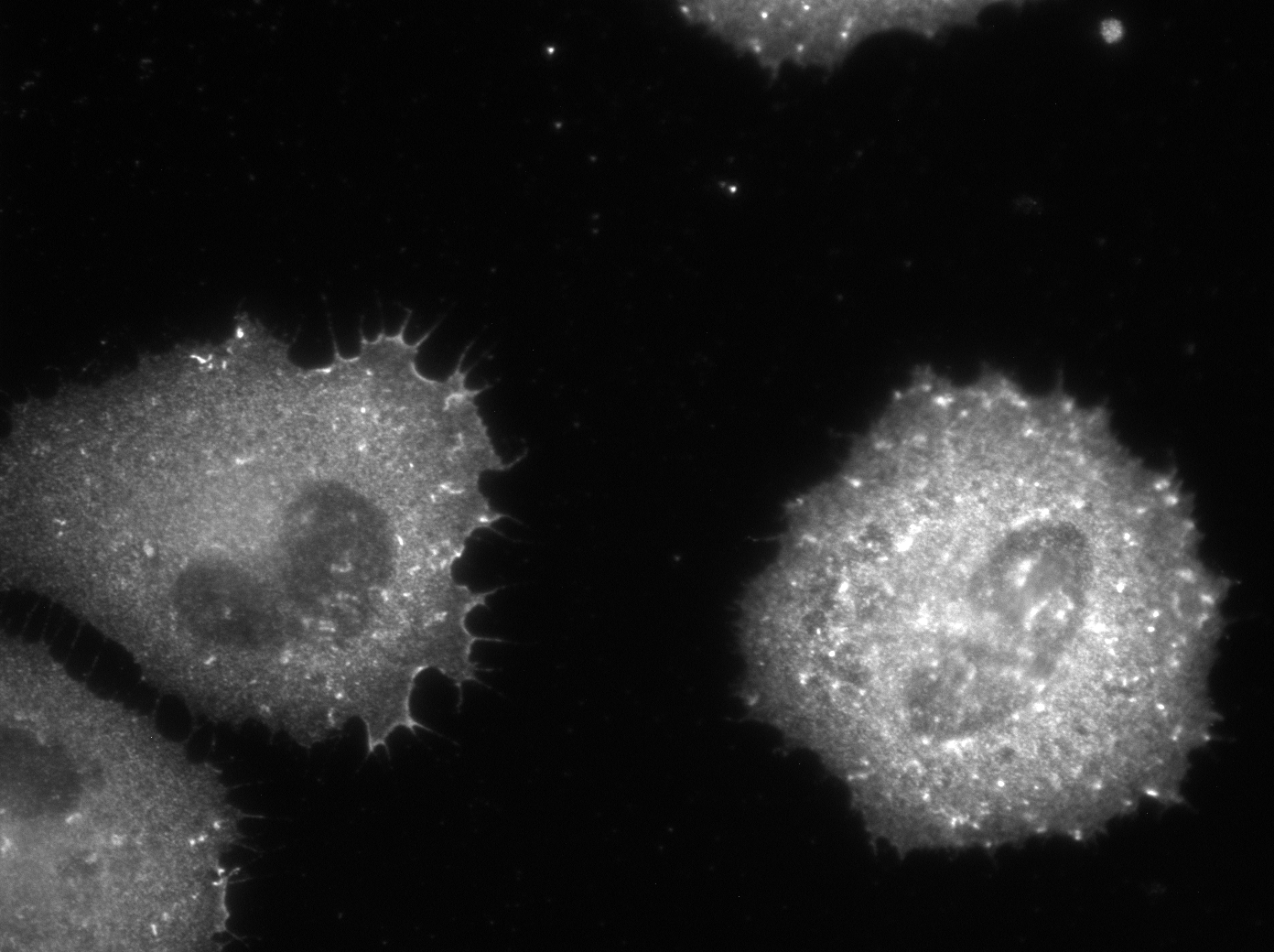

Supplement: Supplementary file 3 — Source data Fig. 1 [file 44319_2024_201_MOESM3_ESM.zip › Figure 1/1B/20210423_HeLa_WR_cortactin ab-488_CK666_200uM.TIF]

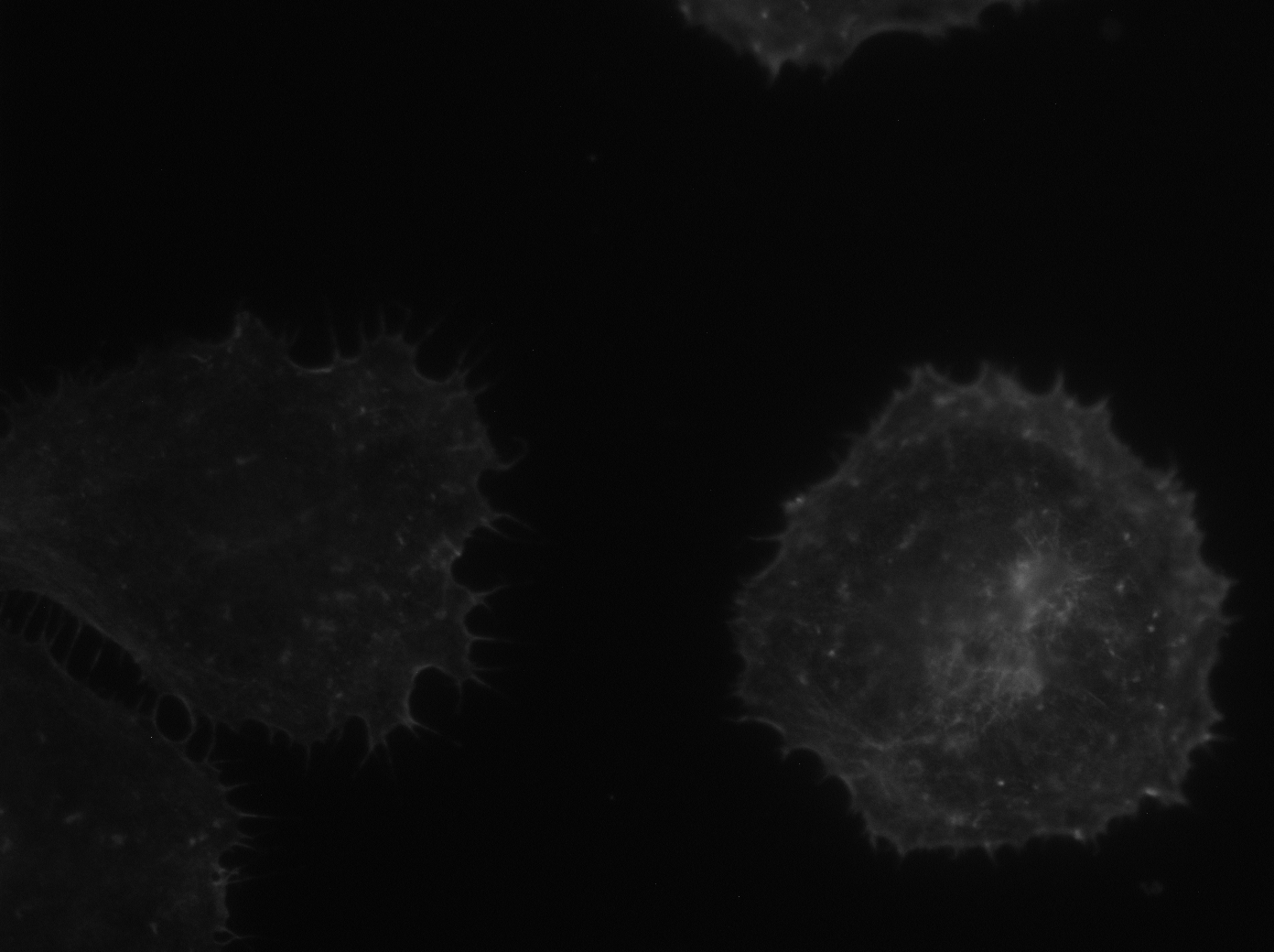

Supplement: Supplementary file 3 — Source data Fig. 1 [file 44319_2024_201_MOESM3_ESM.zip › Figure 1/1B/20210423_HeLa_WR_exB5-647_CK666_200uM.TIF]

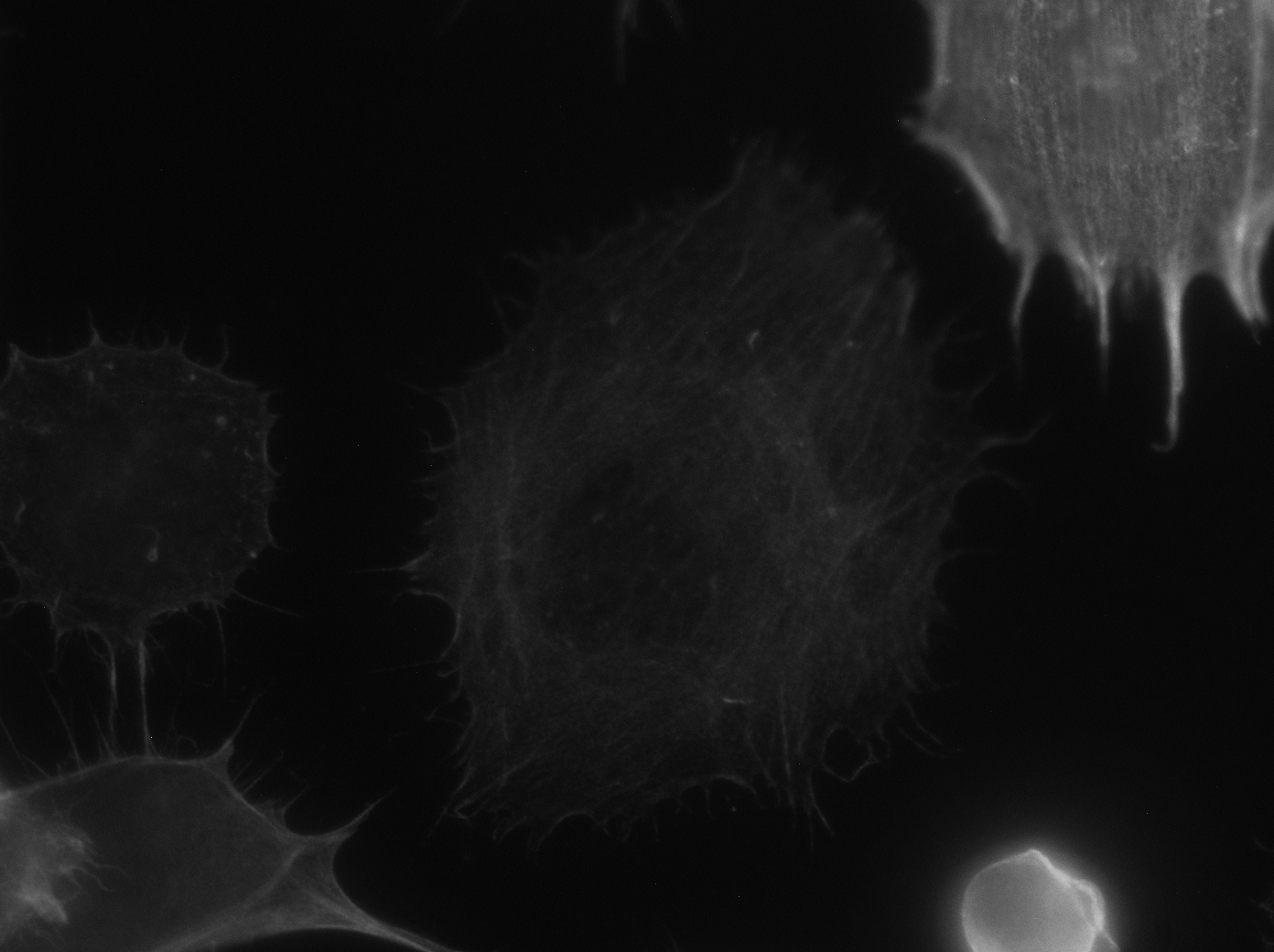

Supplement: Supplementary file 3 — Source data Fig. 1 [file 44319_2024_201_MOESM3_ESM.zip › Figure 1/1B/20210423_HeLa_WR_exB5-647_CK869_100uM.TIF]

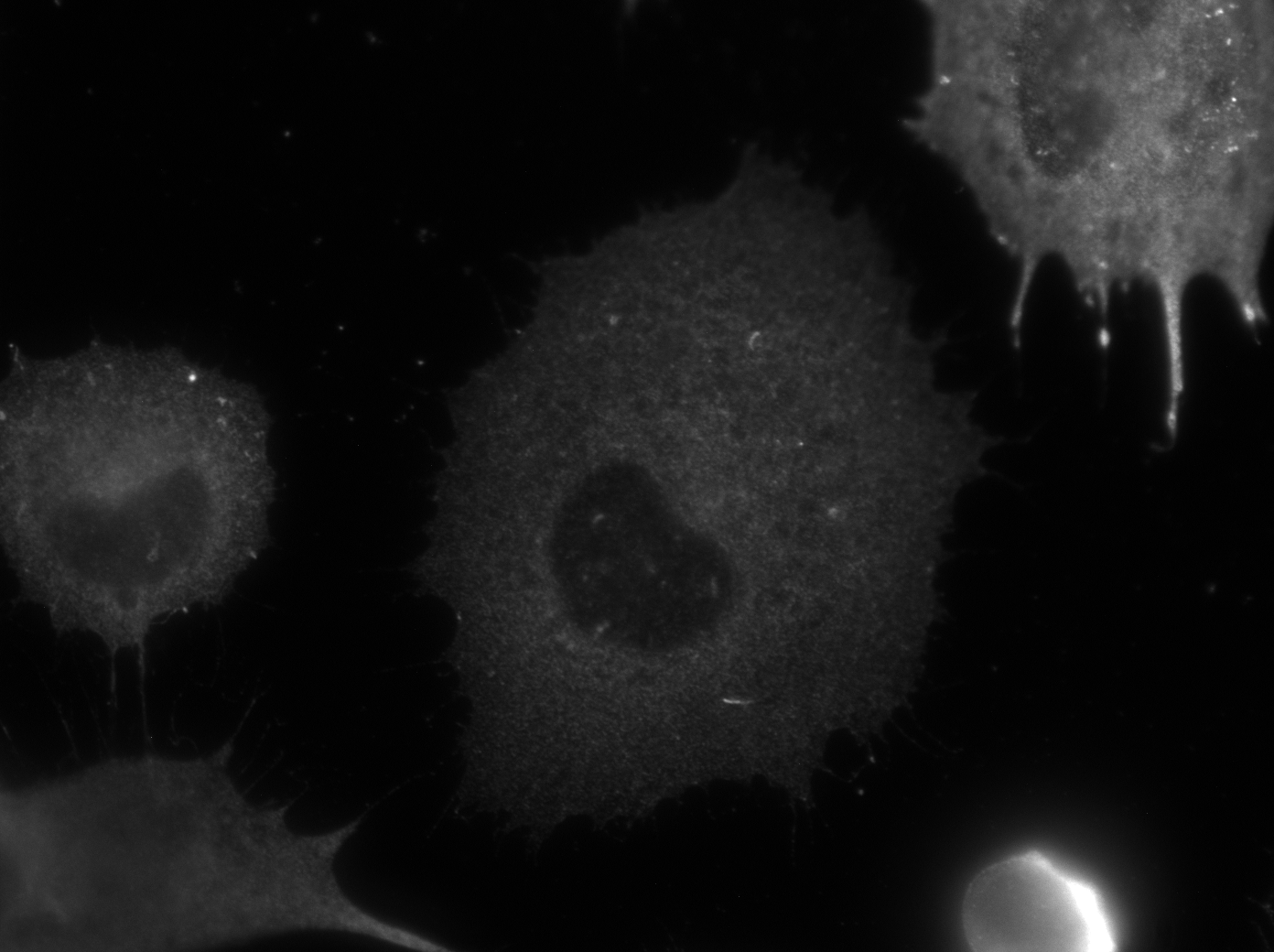

Supplement: Supplementary file 3 — Source data Fig. 1 [file 44319_2024_201_MOESM3_ESM.zip › Figure 1/1B/20210423_HeLa_WR_cortactin ab-488_CK869_100uM.TIF]

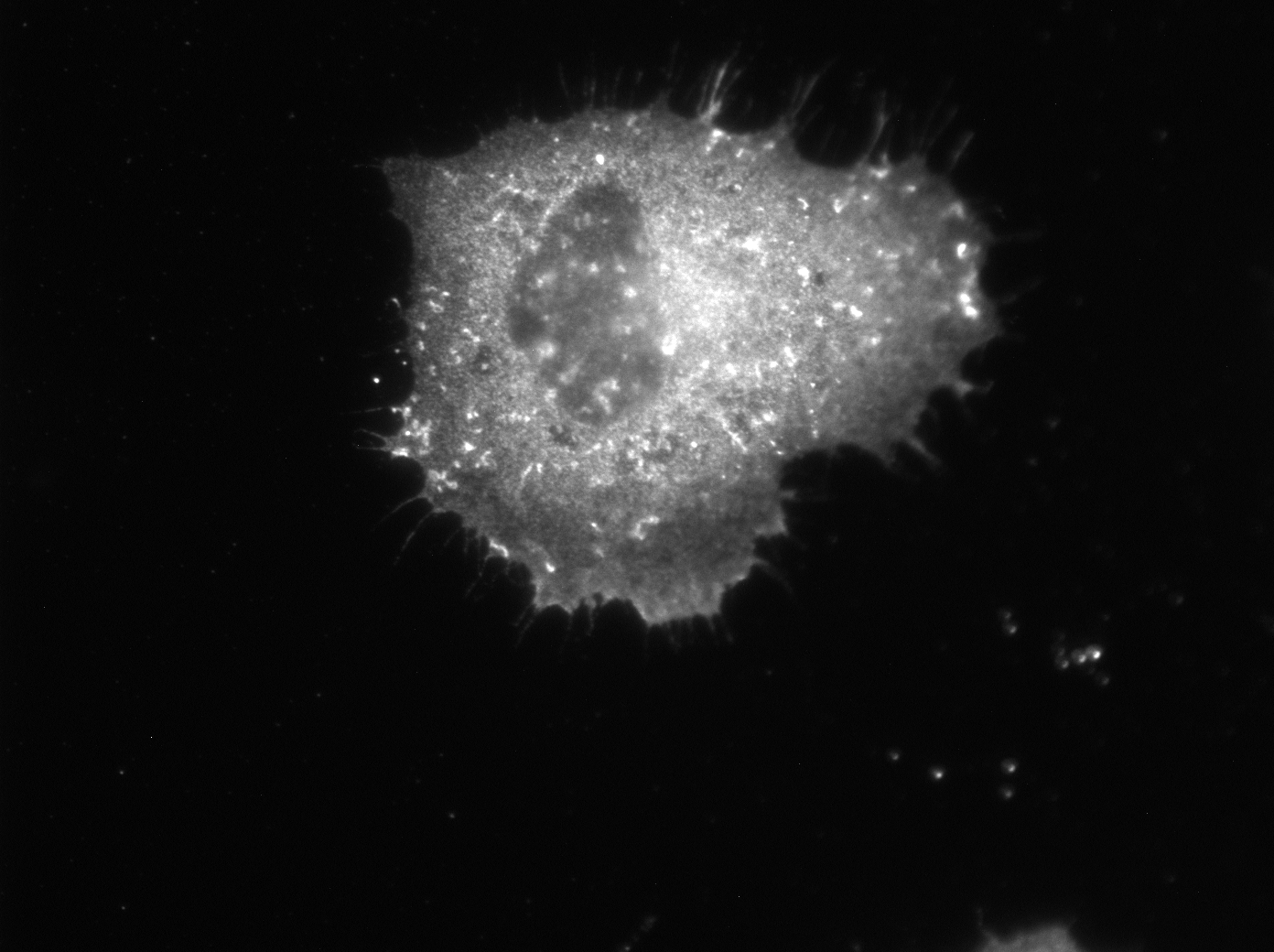

Supplement: Supplementary file 3 — Source data Fig. 1 [file 44319_2024_201_MOESM3_ESM.zip › Figure 1/1B/20210423_HeLa_WR_cortactin ab-488_CK666_300uM.TIF]

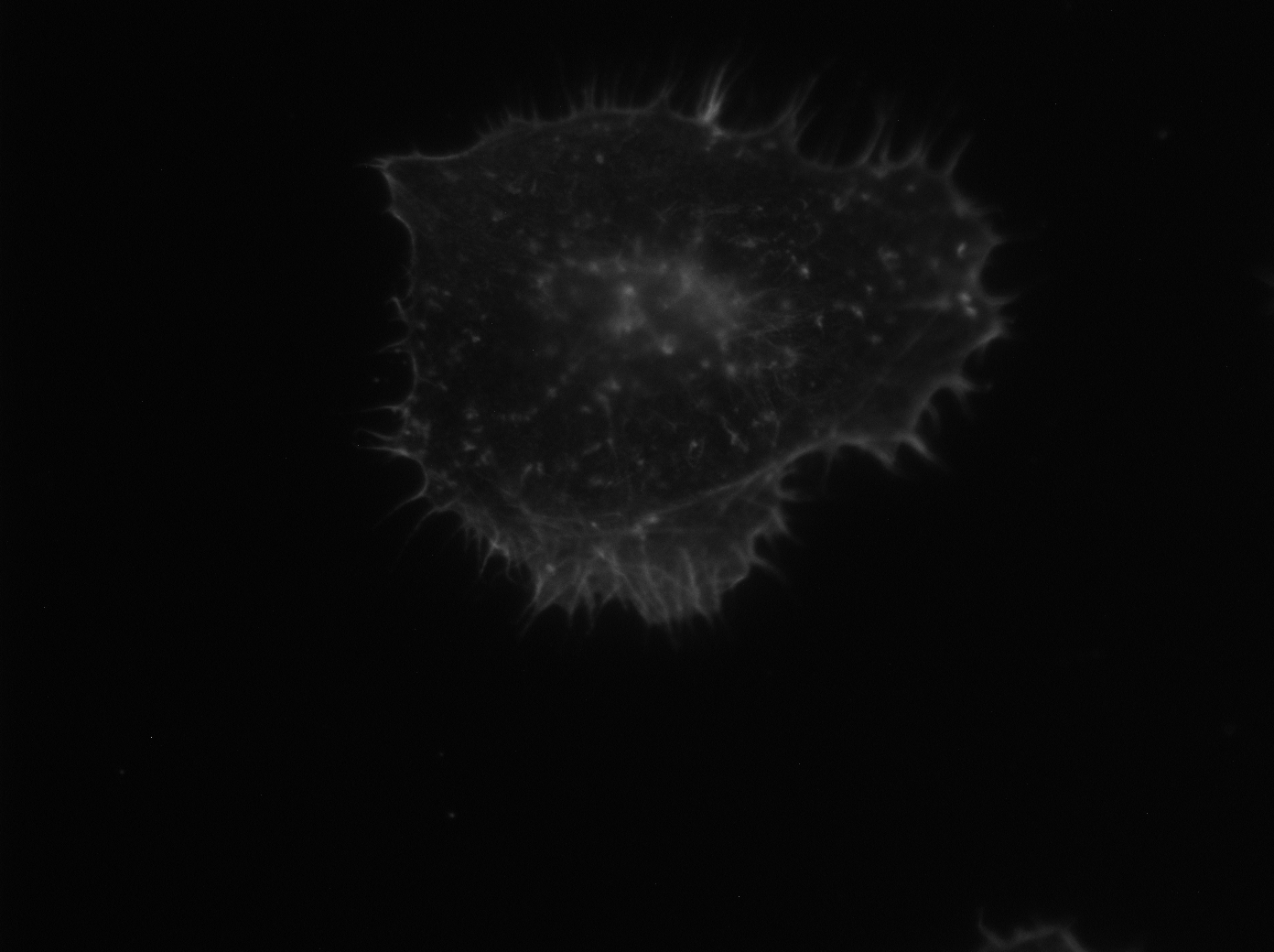

Supplement: Supplementary file 3 — Source data Fig. 1 [file 44319_2024_201_MOESM3_ESM.zip › Figure 1/1B/20210423_HeLa_WR_exB5-647_CK666_300uM.TIF]

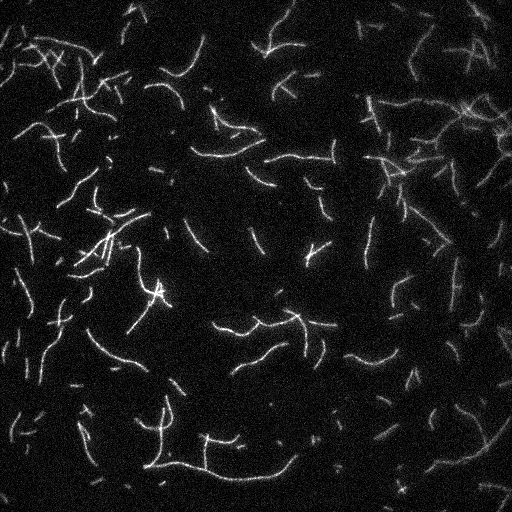

Supplement: Supplementary file 4 — Source data Fig. 2 [file 44319_2024_201_MOESM4_ESM.zip › Figure 2/2B/C1BC5L CK666.tif]

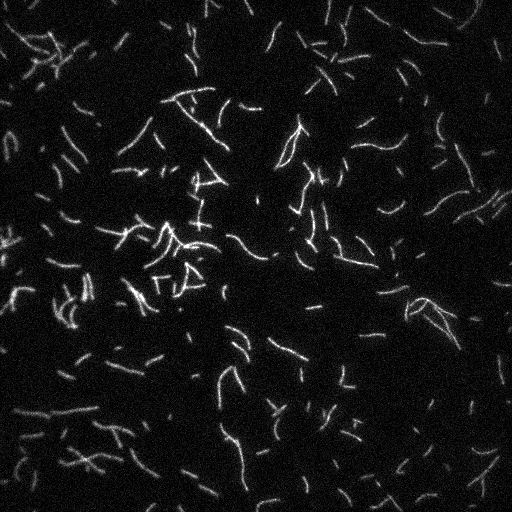

Supplement: Supplementary file 4 — Source data Fig. 2 [file 44319_2024_201_MOESM4_ESM.zip › Figure 2/2B/C1BC5L CK869.tif]

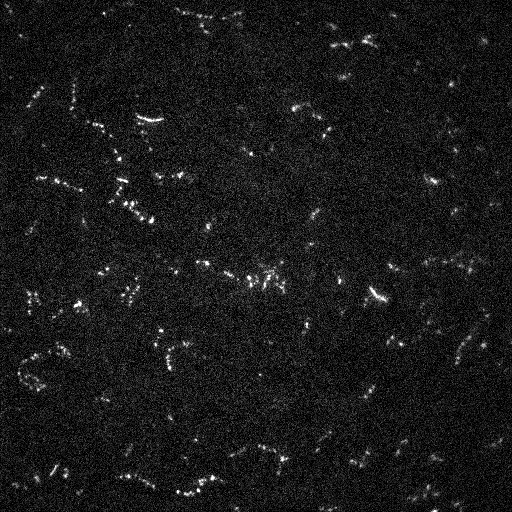

Supplement: Supplementary file 4 — Source data Fig. 2 [file 44319_2024_201_MOESM4_ESM.zip › Figure 2/2B/C1BC5 DMSO.tif]

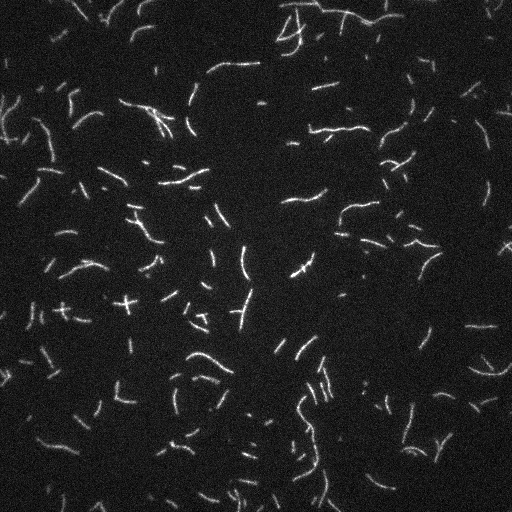

Supplement: Supplementary file 4 — Source data Fig. 2 [file 44319_2024_201_MOESM4_ESM.zip › Figure 2/2B/C1AC5L DMSO.tif]

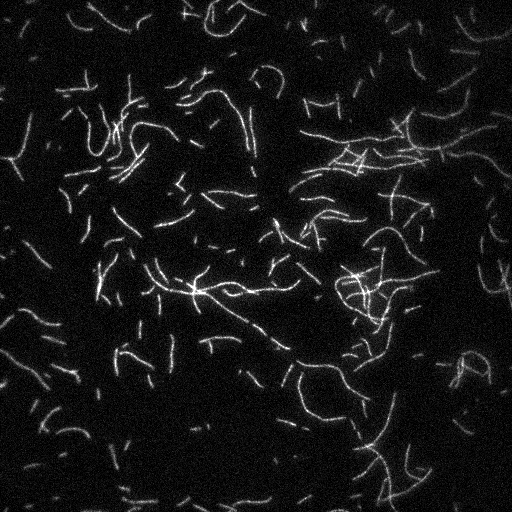

Supplement: Supplementary file 4 — Source data Fig. 2 [file 44319_2024_201_MOESM4_ESM.zip › Figure 2/2B/C1BC5L DMSO.tif]

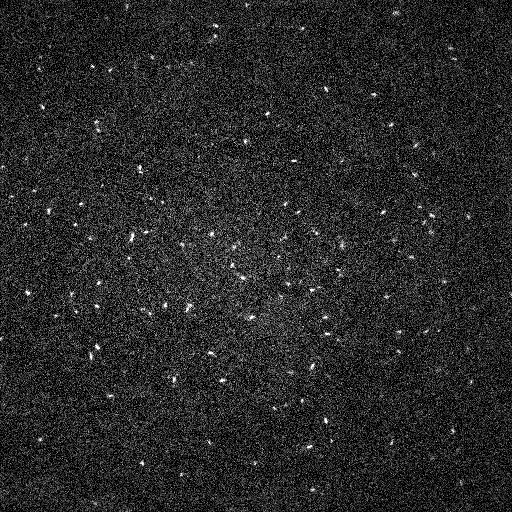

Supplement: Supplementary file 4 — Source data Fig. 2 [file 44319_2024_201_MOESM4_ESM.zip › Figure 2/2B/C1BC5 CK869.tif]

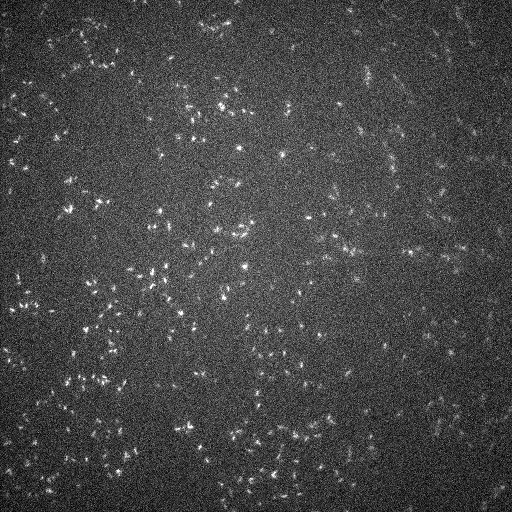

Supplement: Supplementary file 4 — Source data Fig. 2 [file 44319_2024_201_MOESM4_ESM.zip › Figure 2/2B/C1BC5 CK666.tif]

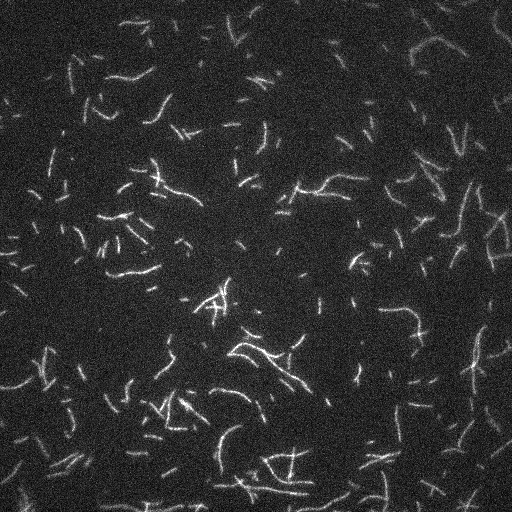

Supplement: Supplementary file 4 — Source data Fig. 2 [file 44319_2024_201_MOESM4_ESM.zip › Figure 2/2B/C1AC5L CK666.tif]

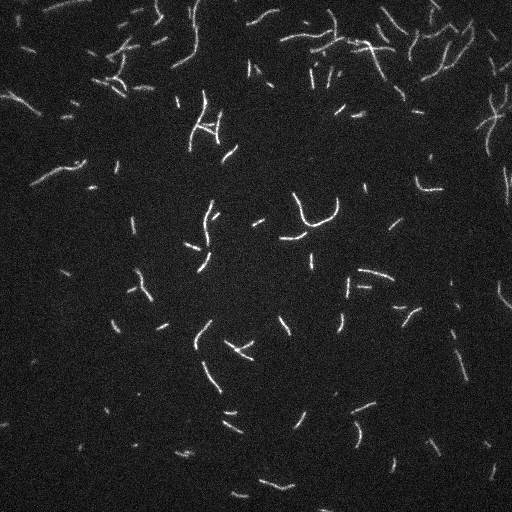

Supplement: Supplementary file 4 — Source data Fig. 2 [file 44319_2024_201_MOESM4_ESM.zip › Figure 2/2B/C1AC5L CK869.tif]

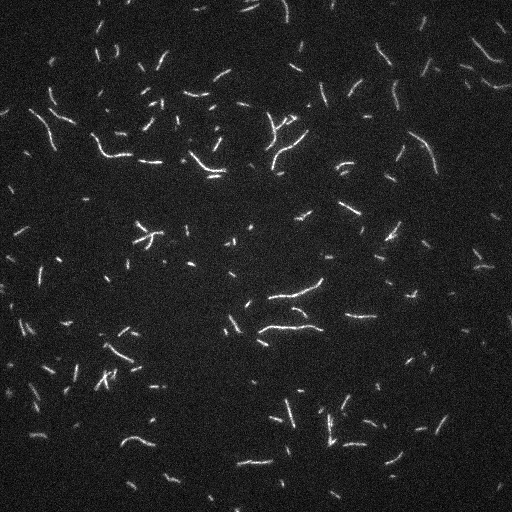

Supplement: Supplementary file 4 — Source data Fig. 2 [file 44319_2024_201_MOESM4_ESM.zip › Figure 2/2B/C1AC5 CK666.tif]

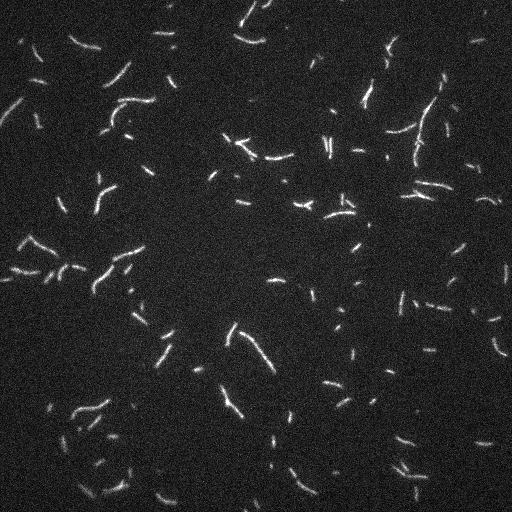

Supplement: Supplementary file 4 — Source data Fig. 2 [file 44319_2024_201_MOESM4_ESM.zip › Figure 2/2B/C1AC5 CK869.tif]

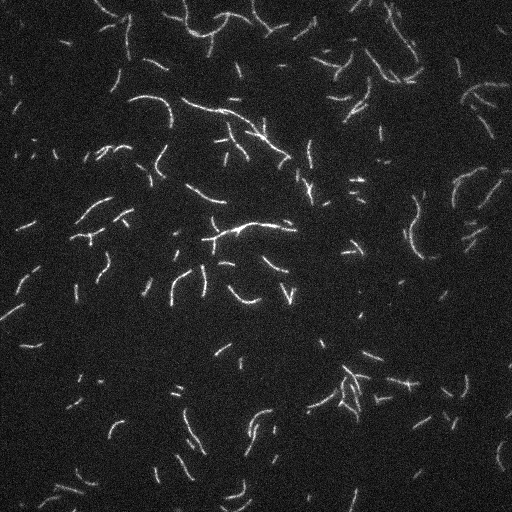

Supplement: Supplementary file 4 — Source data Fig. 2 [file 44319_2024_201_MOESM4_ESM.zip › Figure 2/2B/C1AC5 DMSO.tif]

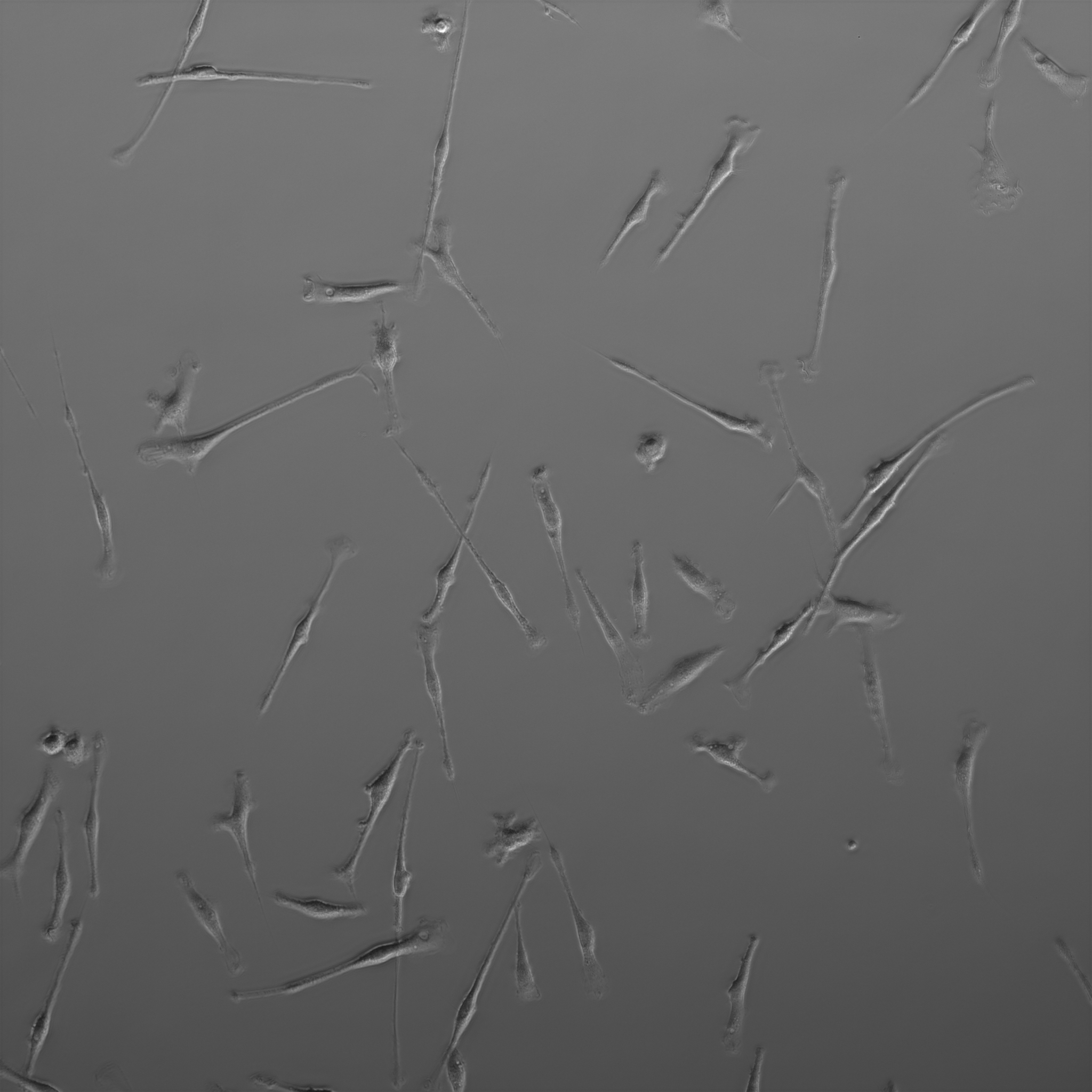

Supplement: Supplementary file 5 — Source data Fig. 3 [file 44319_2024_201_MOESM5_ESM.zip › Figure 3/3B/CK666 10.tif]

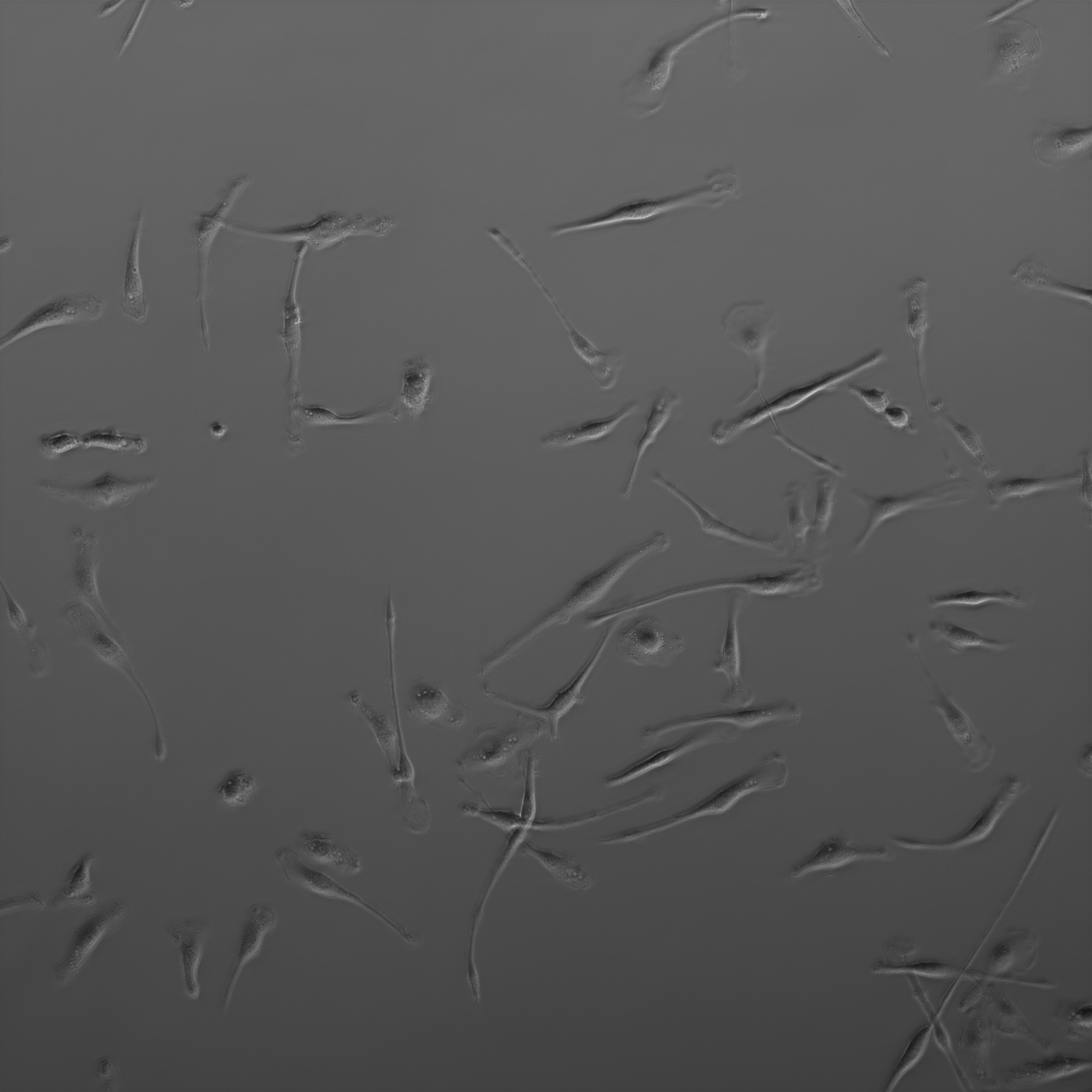

Supplement: Supplementary file 5 — Source data Fig. 3 [file 44319_2024_201_MOESM5_ESM.zip › Figure 3/3B/DMSO 0.tif]

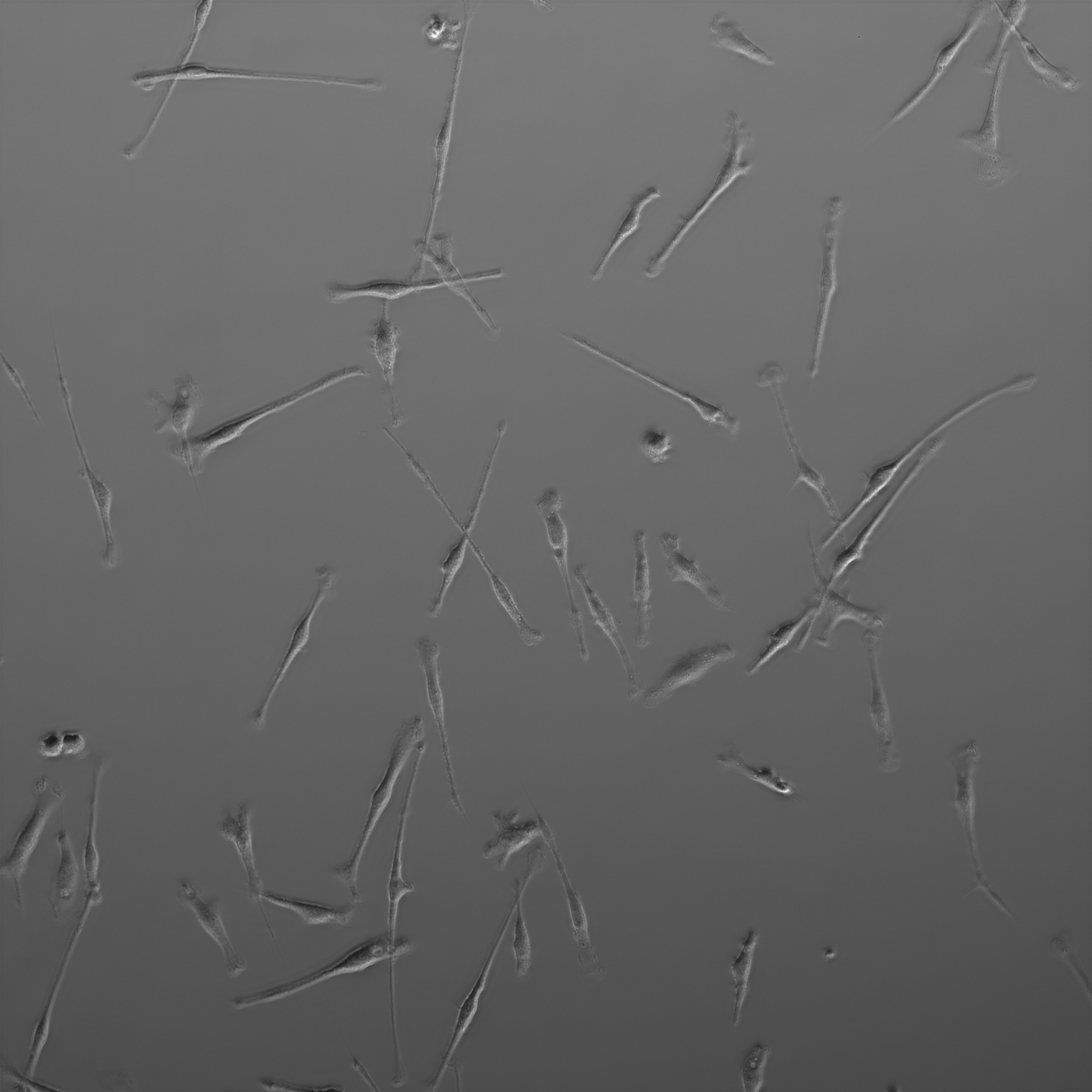

Supplement: Supplementary file 5 — Source data Fig. 3 [file 44319_2024_201_MOESM5_ESM.zip › Figure 3/3B/CK666 0.tif]

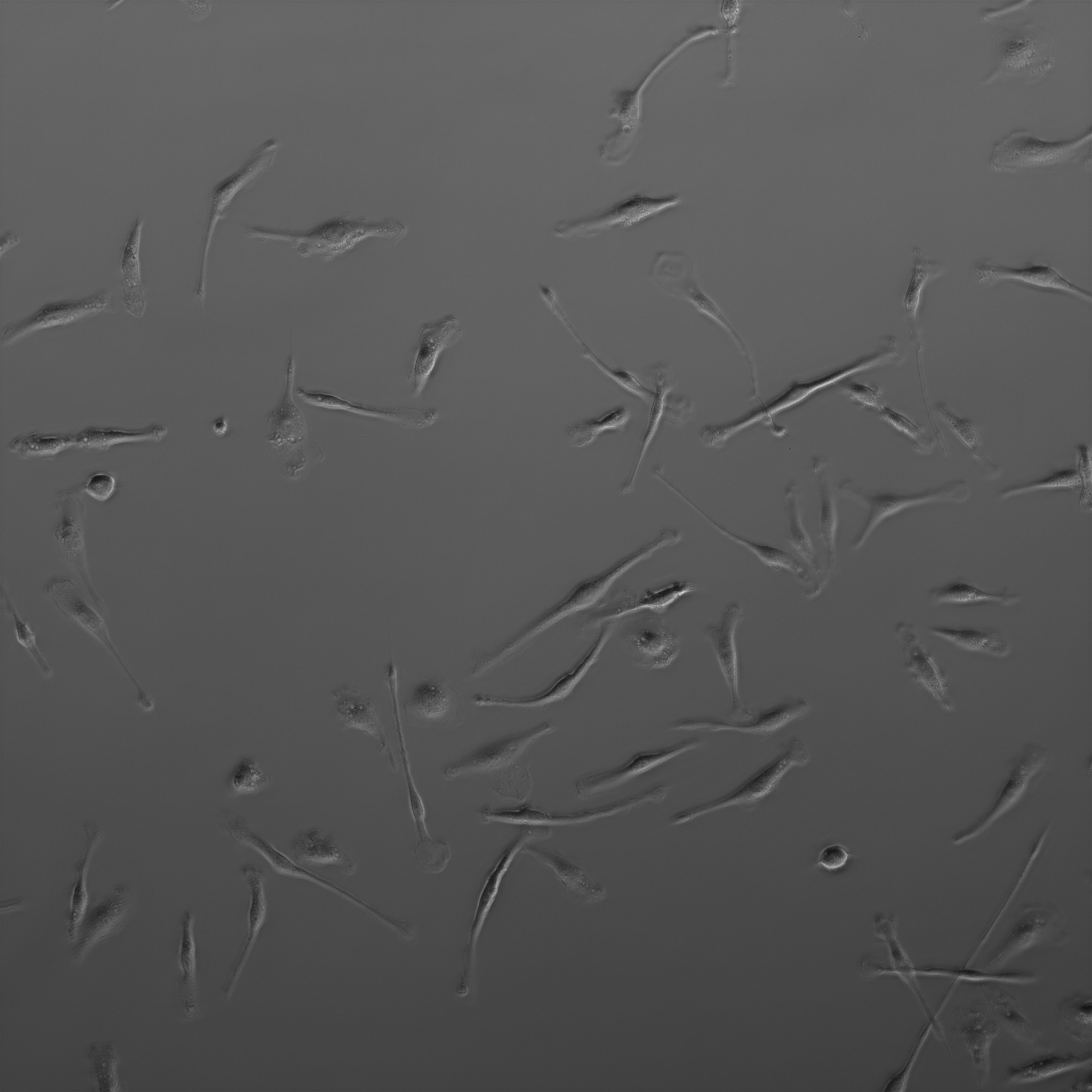

Supplement: Supplementary file 5 — Source data Fig. 3 [file 44319_2024_201_MOESM5_ESM.zip › Figure 3/3B/DMSO 20.tif]

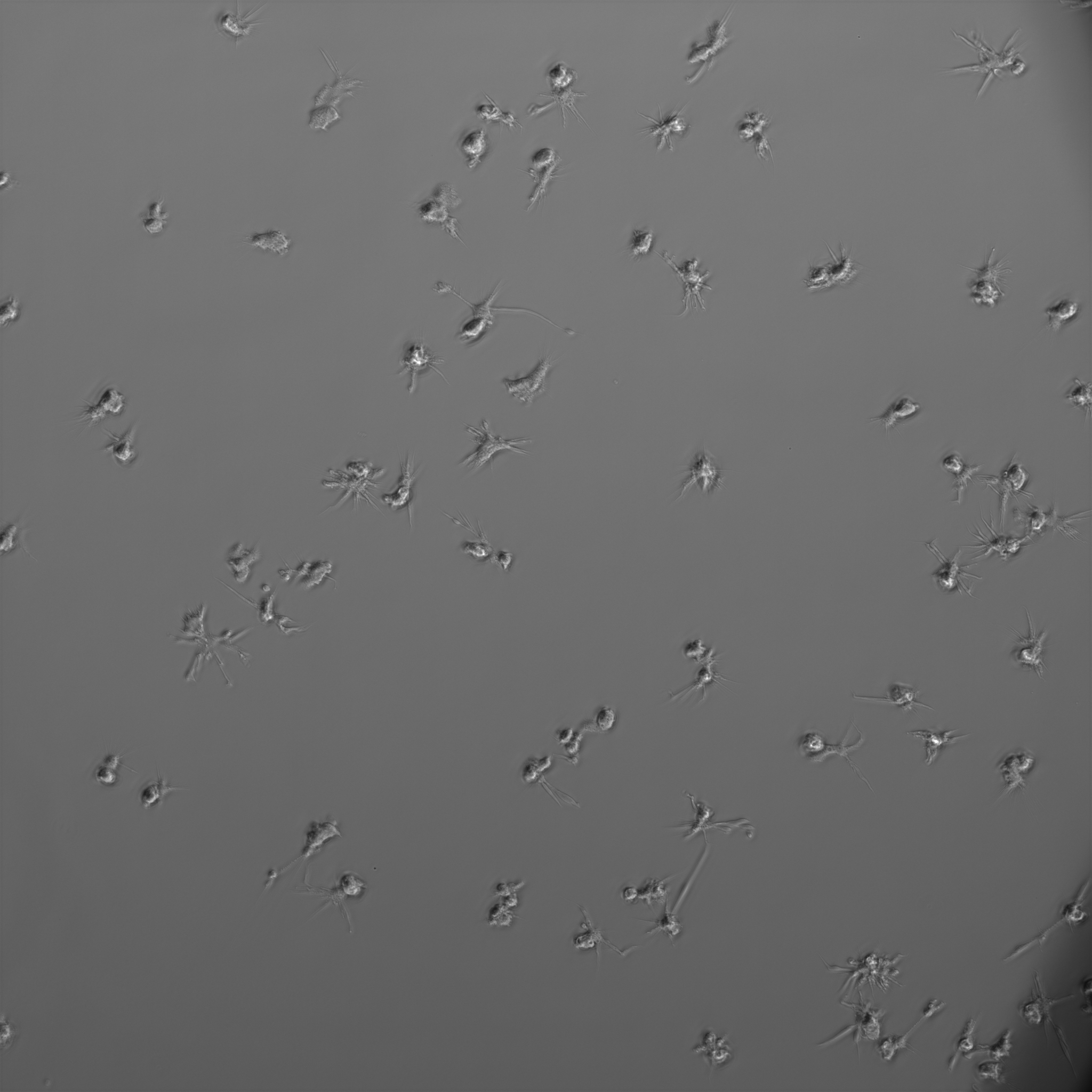

Supplement: Supplementary file 5 — Source data Fig. 3 [file 44319_2024_201_MOESM5_ESM.zip › Figure 3/3B/CK869 10.tif]

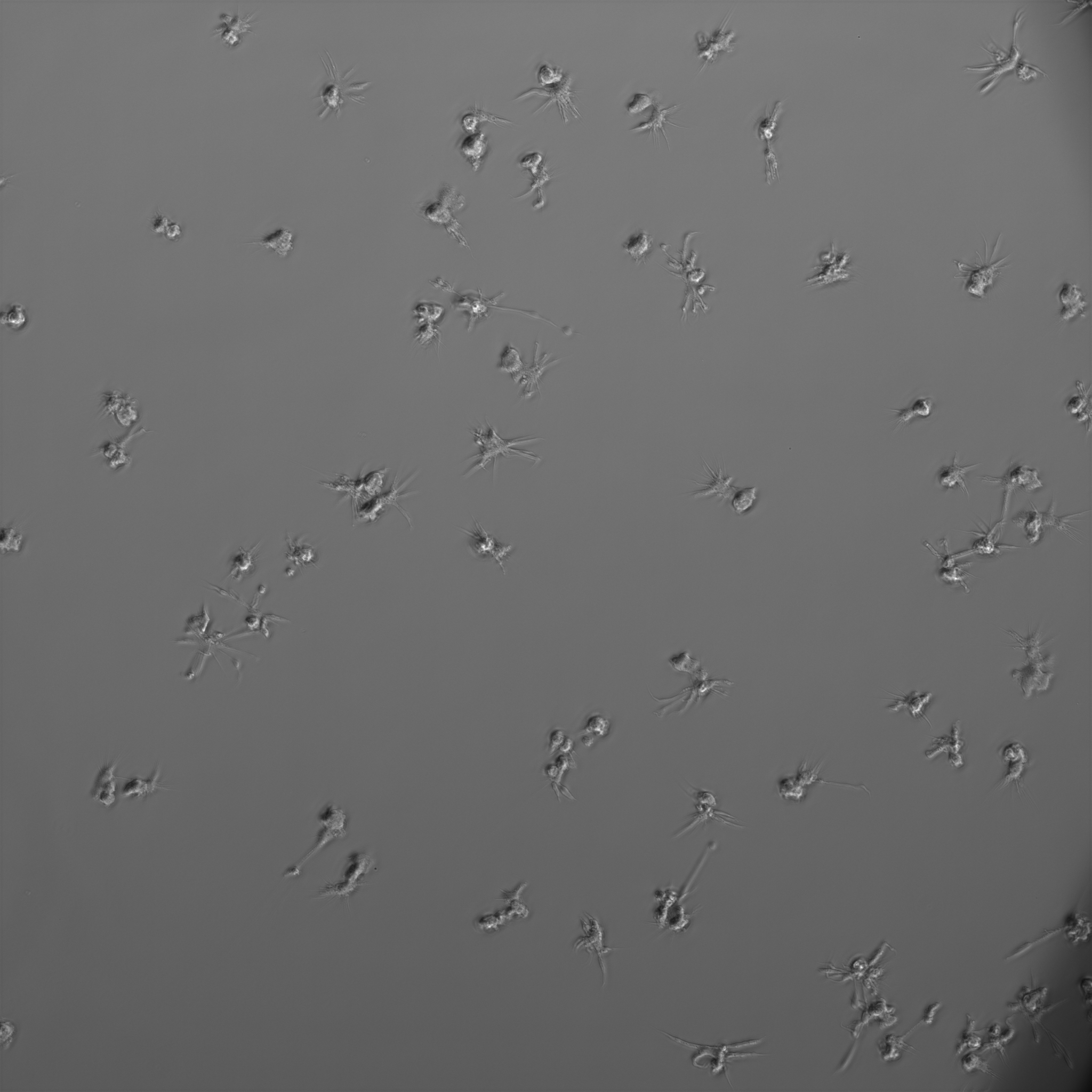

Supplement: Supplementary file 5 — Source data Fig. 3 [file 44319_2024_201_MOESM5_ESM.zip › Figure 3/3B/CK869 0.tif]

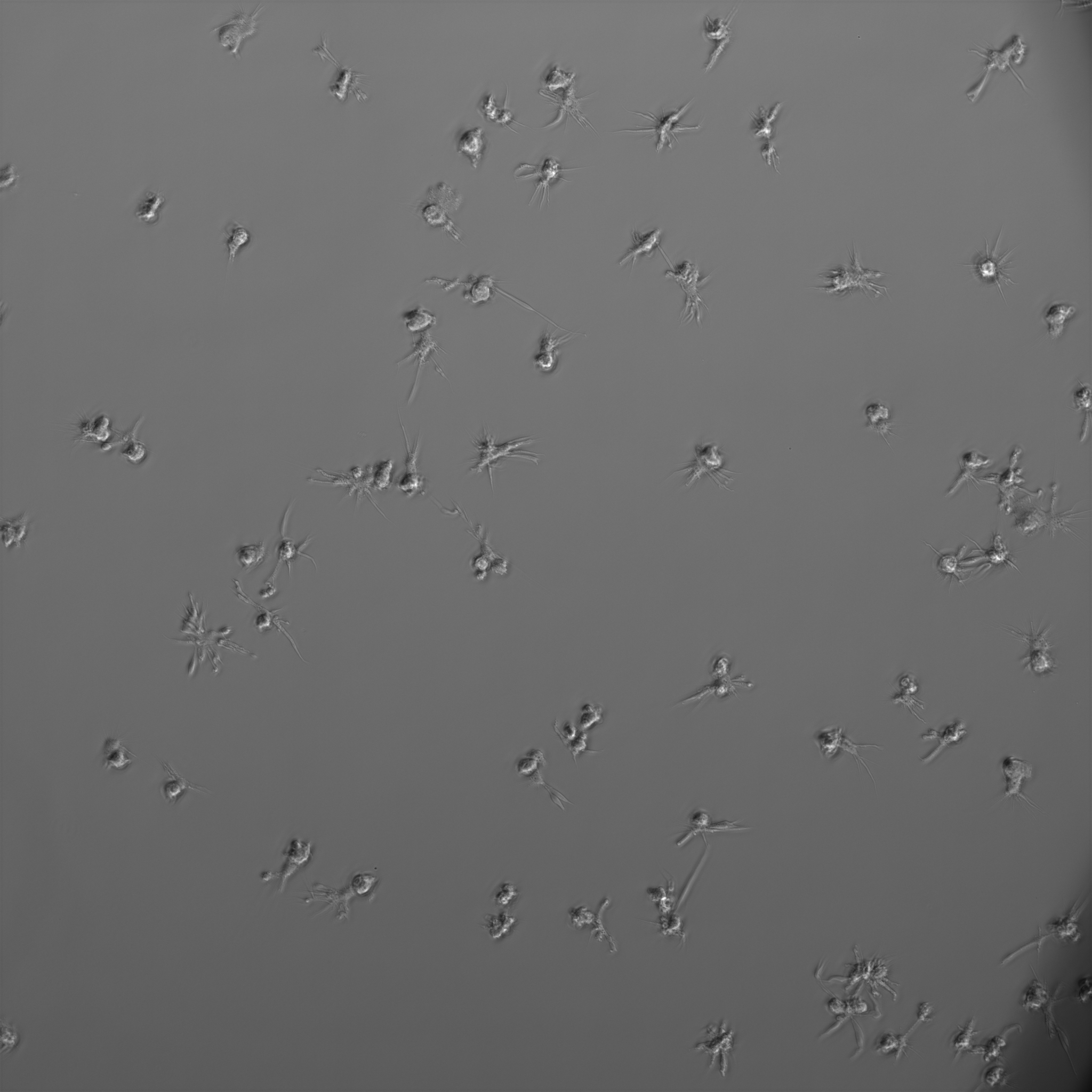

Supplement: Supplementary file 5 — Source data Fig. 3 [file 44319_2024_201_MOESM5_ESM.zip › Figure 3/3B/CK869 20.tif]

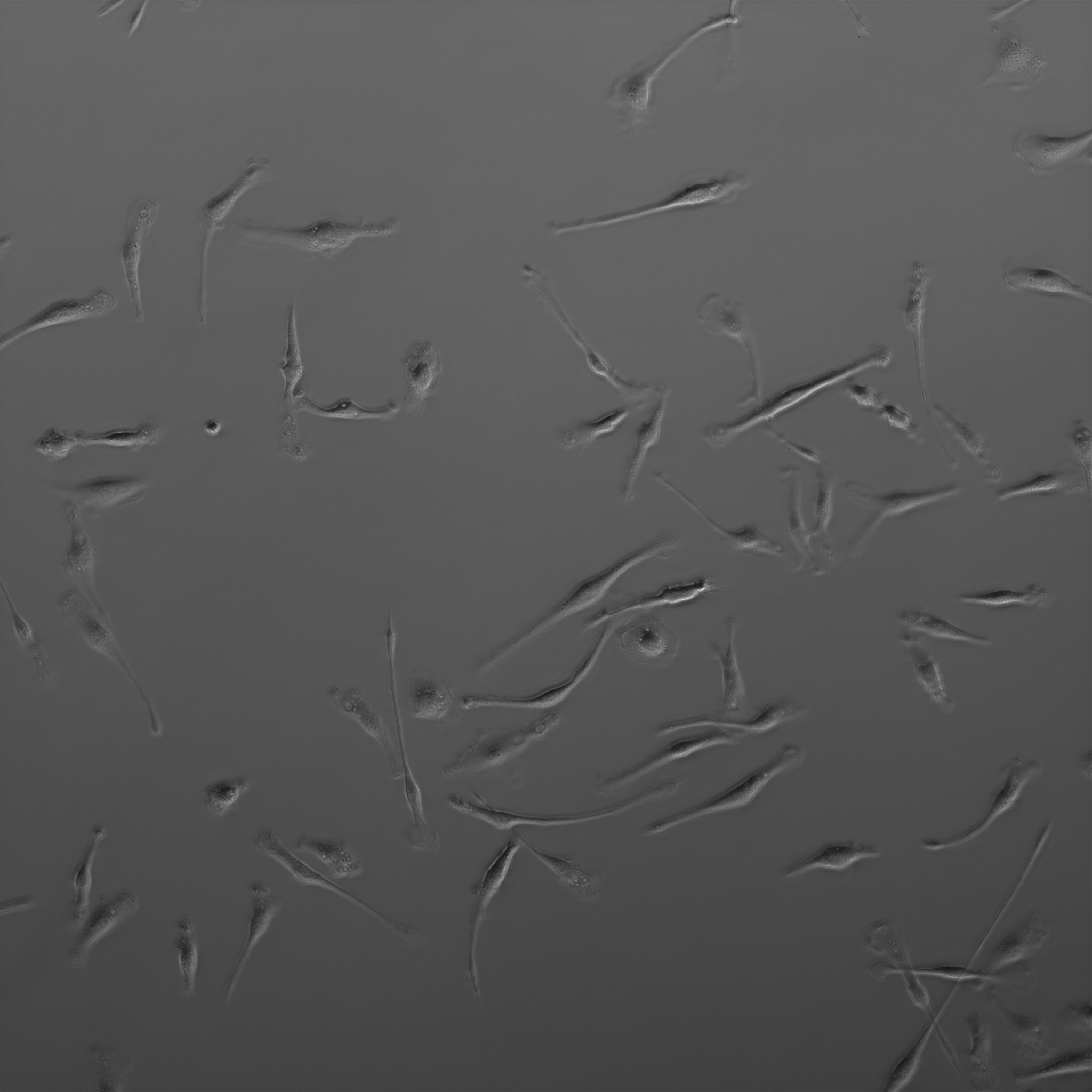

Supplement: Supplementary file 5 — Source data Fig. 3 [file 44319_2024_201_MOESM5_ESM.zip › Figure 3/3B/DMSO 10.tif]

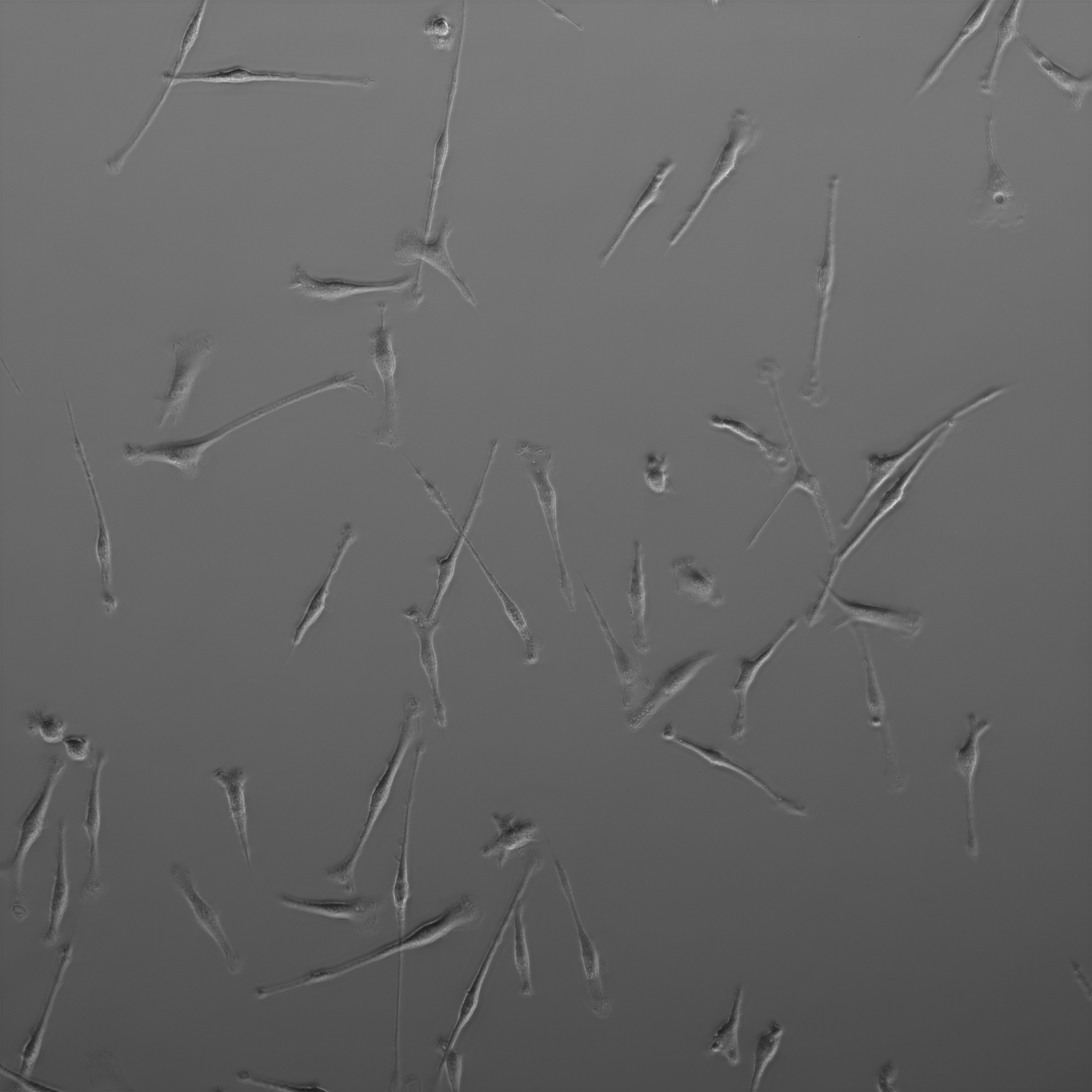

Supplement: Supplementary file 5 — Source data Fig. 3 [file 44319_2024_201_MOESM5_ESM.zip › Figure 3/3B/CK666 20.tif]

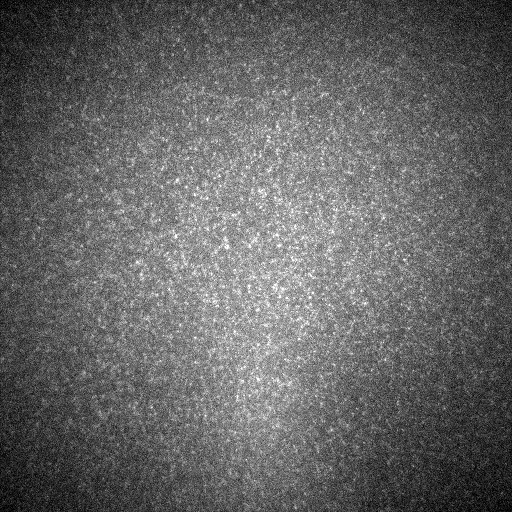

Supplement: Supplementary file 6 — Source data Fig. 4 [file 44319_2024_201_MOESM6_ESM.zip › Figure 4/4B/Figure4B C1A CK666.tif]

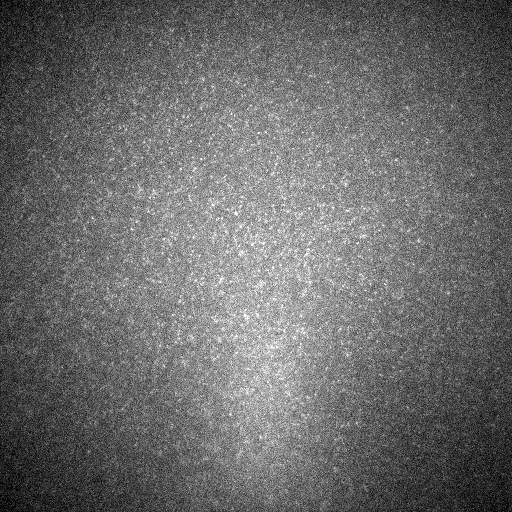

Supplement: Supplementary file 6 — Source data Fig. 4 [file 44319_2024_201_MOESM6_ESM.zip › Figure 4/4B/Figure4B C1A DMSO.tif]

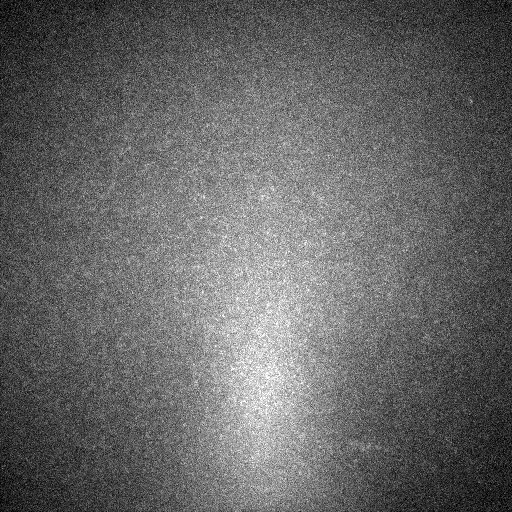

Supplement: Supplementary file 6 — Source data Fig. 4 [file 44319_2024_201_MOESM6_ESM.zip › Figure 4/4B/Figure4B C1A CK869.tif]

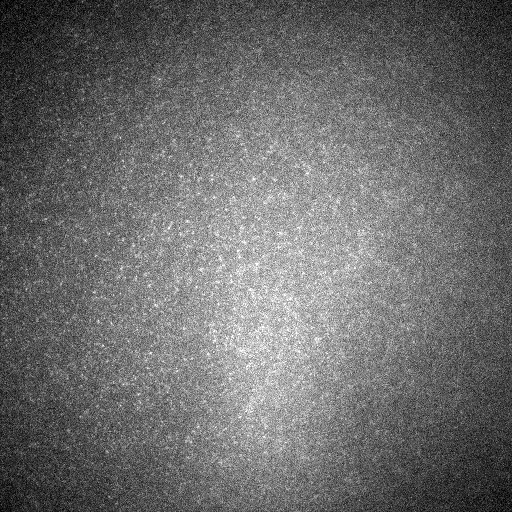

Supplement: Supplementary file 6 — Source data Fig. 4 [file 44319_2024_201_MOESM6_ESM.zip › Figure 4/4B/Figure4B C1B DMSO.tif]

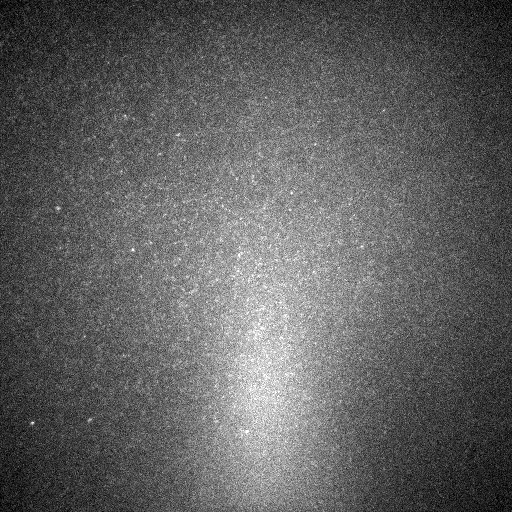

Supplement: Supplementary file 6 — Source data Fig. 4 [file 44319_2024_201_MOESM6_ESM.zip › Figure 4/4B/Figure4B C1B CK869.tif]

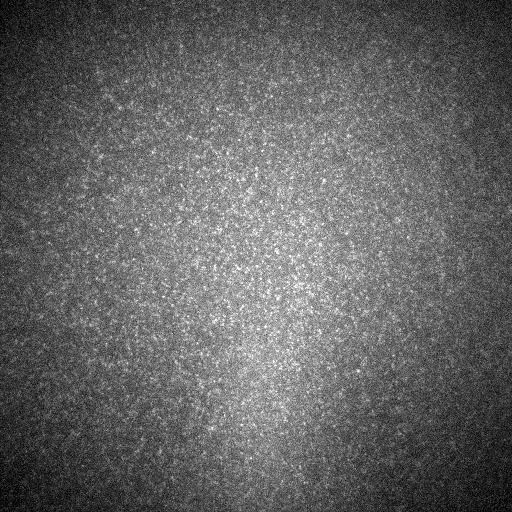

Supplement: Supplementary file 6 — Source data Fig. 4 [file 44319_2024_201_MOESM6_ESM.zip › Figure 4/4B/Figure4B C1B CK666.tif]

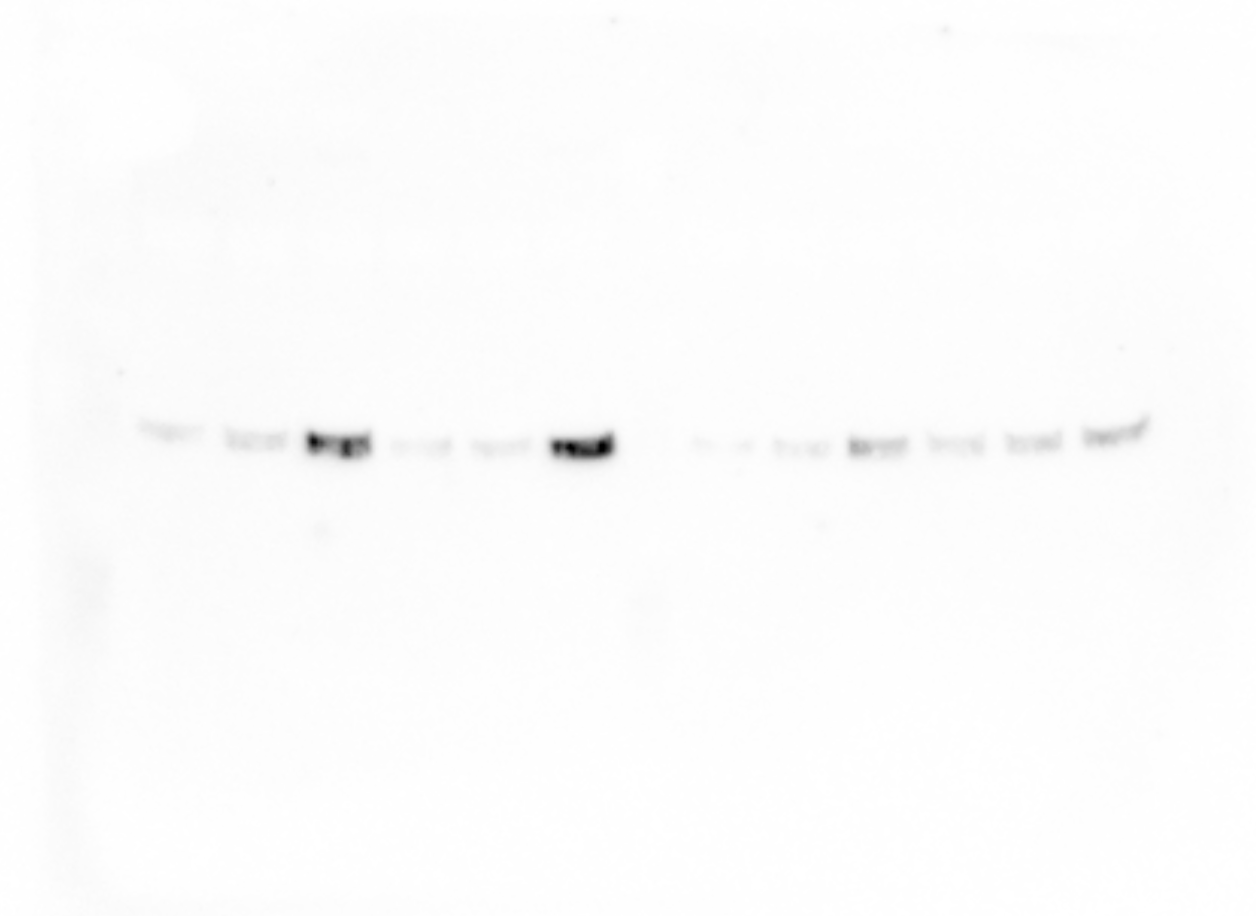

Supplement: Supplementary file 6 — Source data Fig. 4 [file 44319_2024_201_MOESM6_ESM.zip › Figure 4/4C/Figure5C.tif]

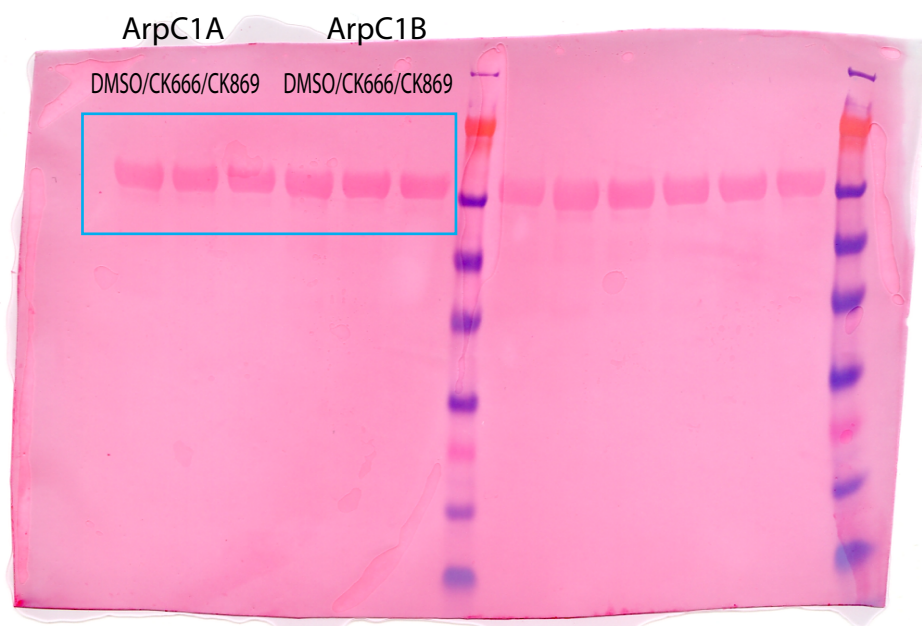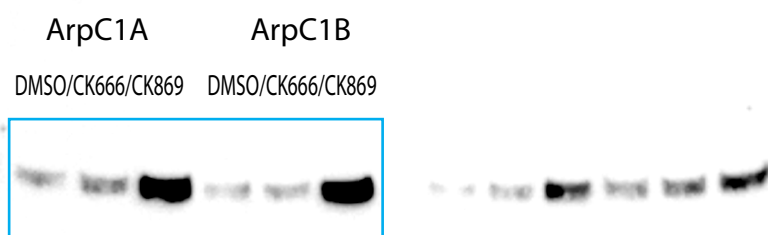

Supplement: Supplementary file 6 — Source data Fig. 4 [file 44319_2024_201_MOESM6_ESM.zip › Figure 4/4C/Figure5C README.pdf]

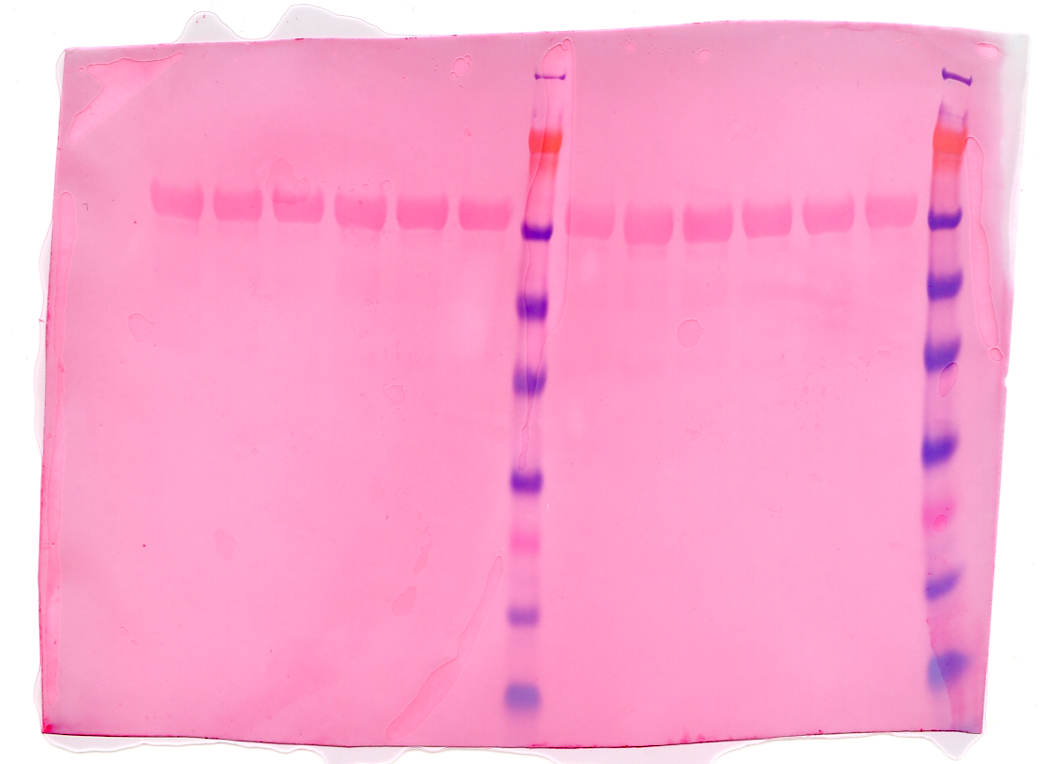

Supplement: Supplementary file 6 — Source data Fig. 4 [file 44319_2024_201_MOESM6_ESM.zip › Figure 4/4C/Figure5C ponceau.tif]

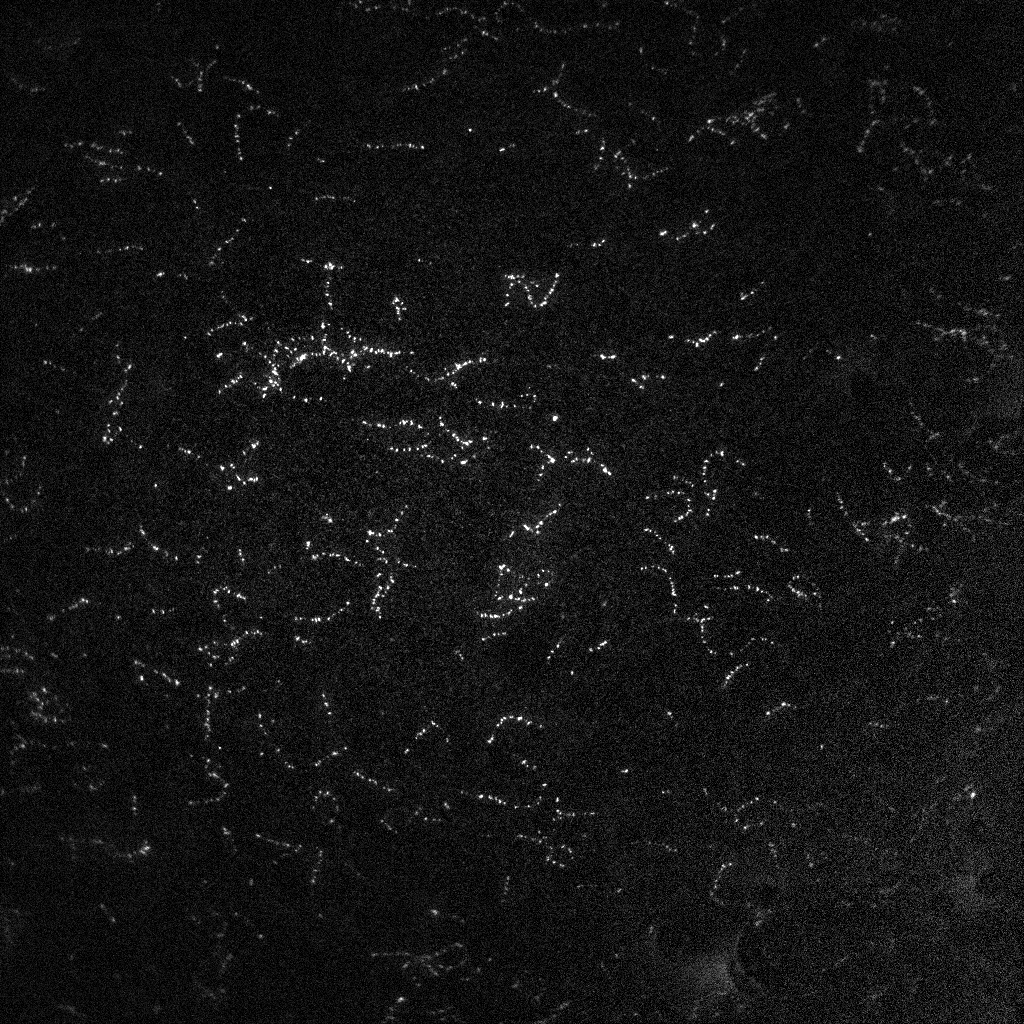

Supplement: Supplementary file 7 — Source data Fig. 5 [file 44319_2024_201_MOESM7_ESM.zip › Figure 5/5A/Figure5A CK869.tif]

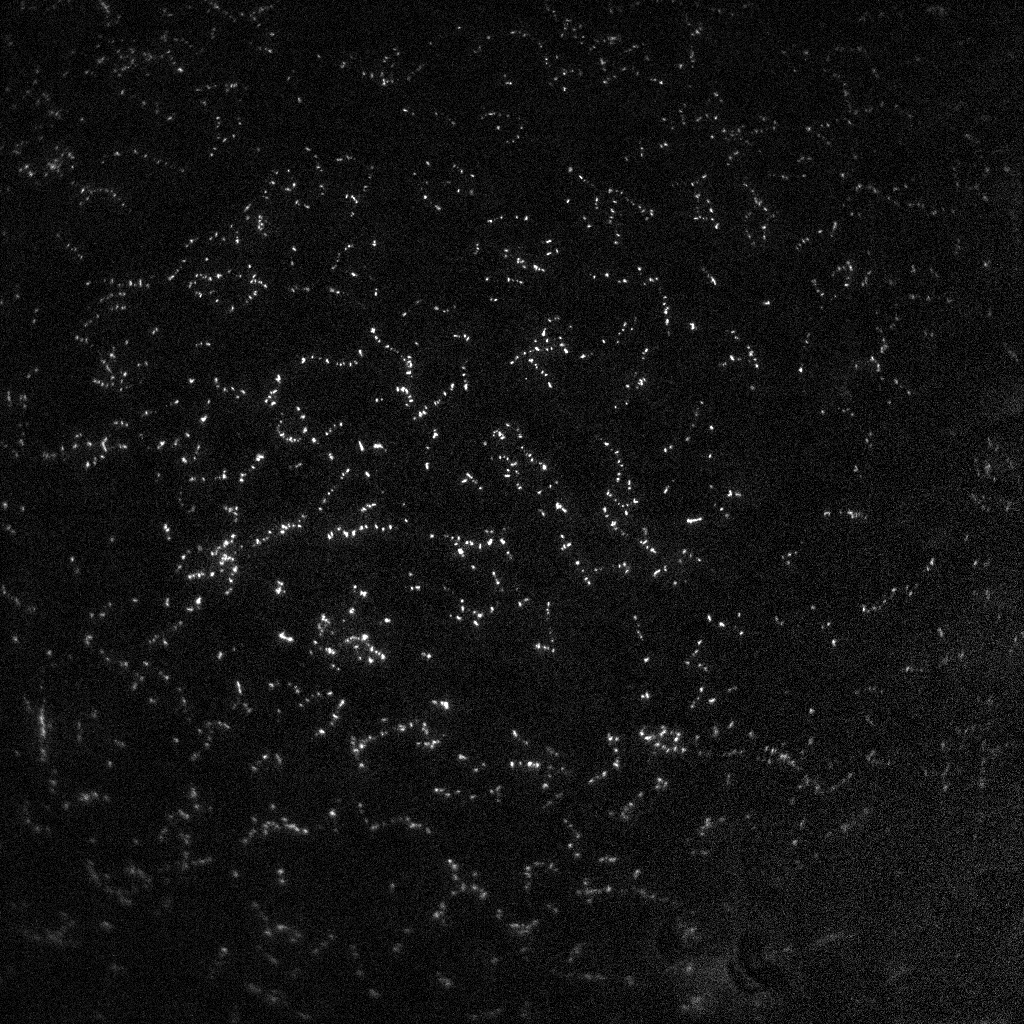

Supplement: Supplementary file 7 — Source data Fig. 5 [file 44319_2024_201_MOESM7_ESM.zip › Figure 5/5A/Figure5A CK666.tif]

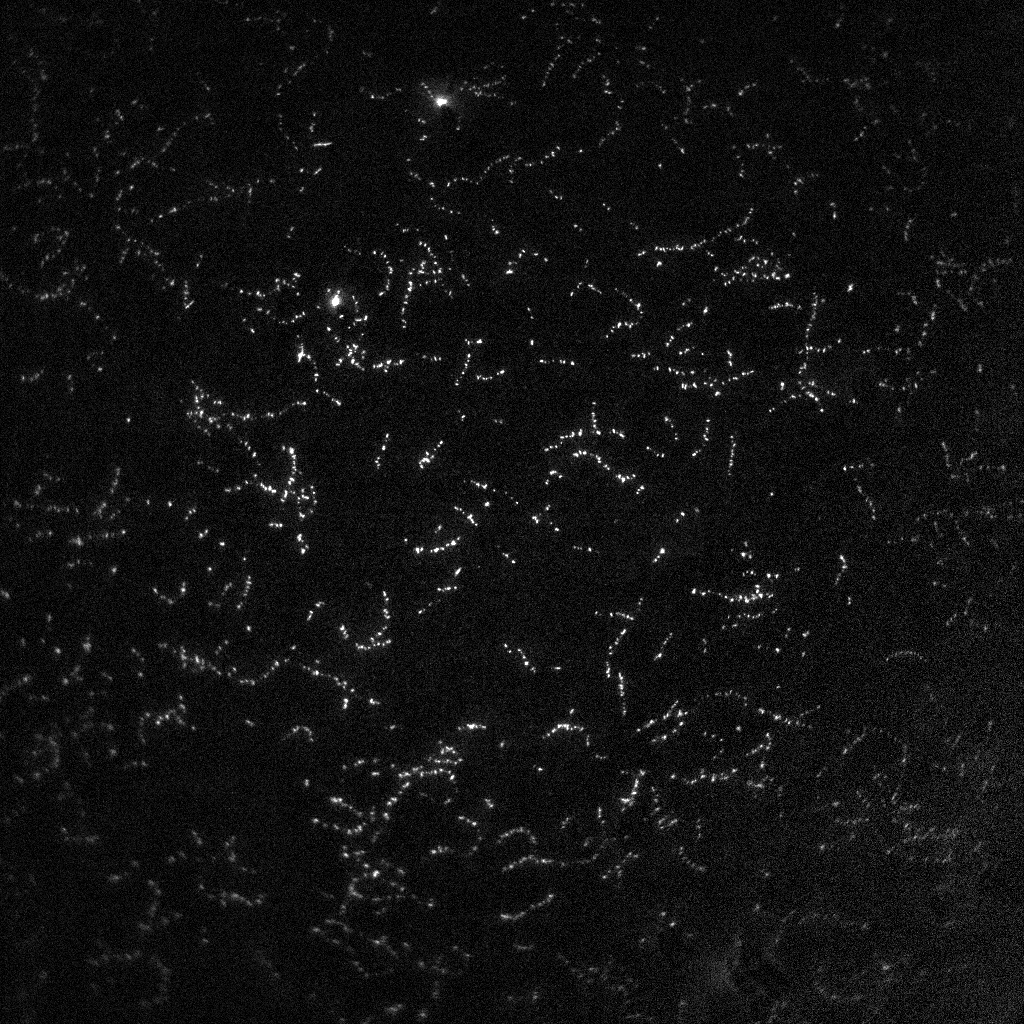

Supplement: Supplementary file 7 — Source data Fig. 5 [file 44319_2024_201_MOESM7_ESM.zip › Figure 5/5A/Figure5A DMSO.tif]

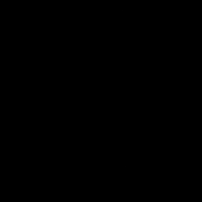

Supplement: Supplementary file 7 — Source data Fig. 5 [file 44319_2024_201_MOESM7_ESM.zip › Figure 5/5B/Figure5B DMSO.tif]

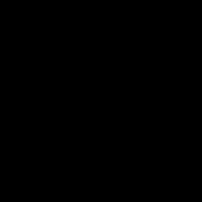

Supplement: Supplementary file 7 — Source data Fig. 5 [file 44319_2024_201_MOESM7_ESM.zip › Figure 5/5B/Figure5B CK666.tif]

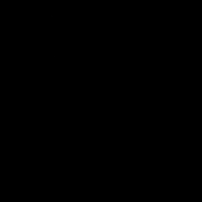

Supplement: Supplementary file 7 — Source data Fig. 5 [file 44319_2024_201_MOESM7_ESM.zip › Figure 5/5B/Figure5B CK869.tif]
