# Supplementary material for: CK-666 and CK-869 differentially inhibit Arp2/3 iso-complexes
Source: EMBO Rep. 2024 Jul 15;25(8):7. doi: 10.1038/s44319-024-00201-x (PMC11316031; doi:10.1038/s44319-024-00201-x)
Supplement: Supplementary file 6 — Source data Fig. 4 [file 44319_2024_201_MOESM6_ESM.zip › Figure 4/4B/Figure 4B README.pdf]

Figure 4B
Arp2/3, SPIN90 and G-actin were mixed (Time 0) and loaded on the microscope with a frame rate 10 s/ img.

Figure 4B C1A DMSO.tif
Start time: 1min*.
*It takes 1 min from mixing everything to the first img required. So that the first img in the tif file is 60s after the experiment started.

Figure 4B C1A CK-666.tif
Start time: 1min 50s

Figure 4B C1A CK-869.tif
Start time: 1min 30s
Figure 4B C1B DMSO.tif
Start time: 1min
Figure 4B C1B CK-666.tif
Start time: 1min
Figure 4B C1B CK-869.tif
Start time: 1min
