# Supplementary material for: CK-666 and CK-869 differentially inhibit Arp2/3 iso-complexes
Source: EMBO Rep. 2024 Jul 15;25(8):7. doi: 10.1038/s44319-024-00201-x (PMC11316031; doi:10.1038/s44319-024-00201-x)
Supplement: Supplementary file 7 — Source data Fig. 5 [file 44319_2024_201_MOESM7_ESM.zip › Figure 5/5B/Figure5B README.pdf]

Arp2/3, SPIN90 and G-actin were mixed (Time 0) and loaded on the microscope with a frame rate 10 s/ img.

SPIN90 activation

1. DMSO
   Start time 1 min 40s

2. CK-666
   Start time 1 min 50s

3. CK-869
   Start time 50s
