# Supplementary material for: CK-666 and CK-869 differentially inhibit Arp2/3 iso-complexes
Source: EMBO Rep. 2024 Jul 15;25(8):7. doi: 10.1038/s44319-024-00201-x (PMC11316031; doi:10.1038/s44319-024-00201-x)
Supplement: Supplementary file 8 — Expanded View Figures [file 44319_2024_201_MOESM8_ESM.pdf]

Expanded View Figures

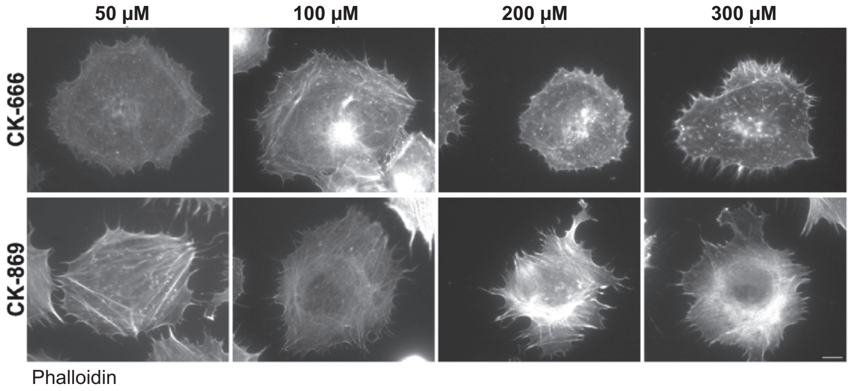

Figure EV1. CK-869, but not CK-666, fully inhibits Vaccinia-induced actin polymerisation.

Immunofluorescence images of the actin cytoskeleton (visualised with phalloidin) of HeLa cells infected with Vaccinia virus at 9 h post-infection after 1-hour incubation with indicated concentrations of CK-666 and CK-869. The images correspond to the actin cytoskeleton of the cortactin images in Fig. 1B. Scale bar = 10 μm.

 © The Author(s)

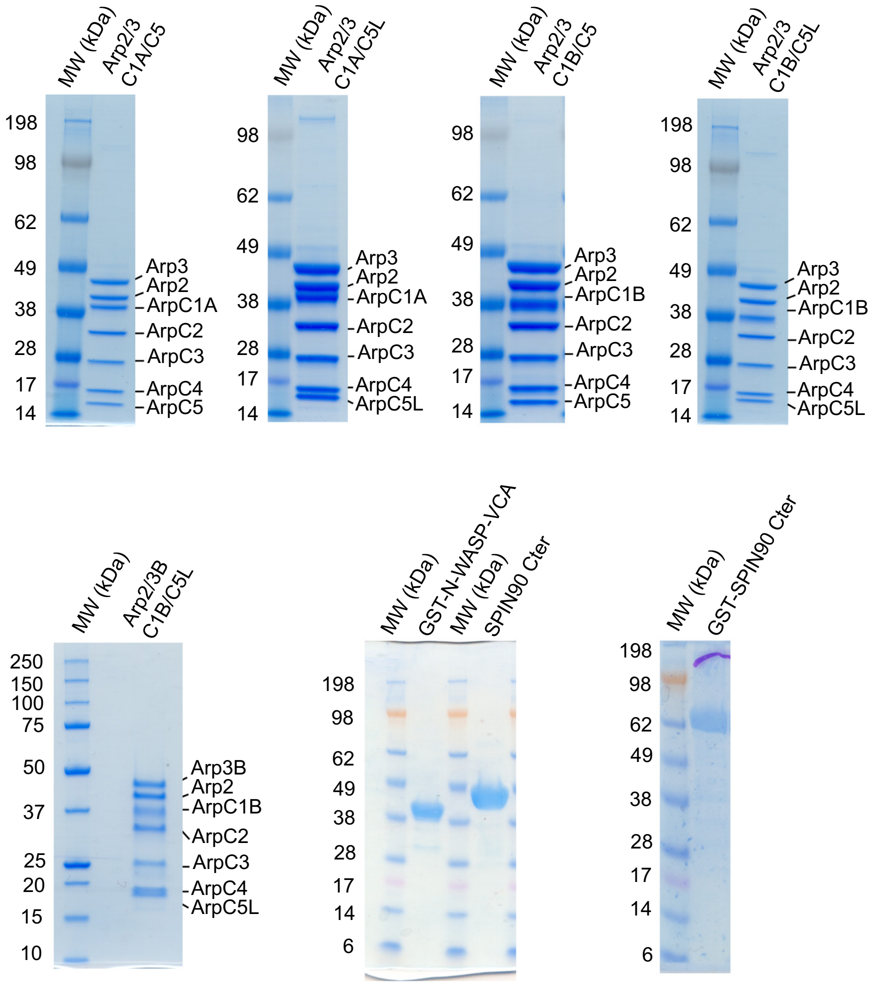

Figure EV2. Coomassie-stained protein gels of the recombinant proteins and Arp2/3 iso-complexes.

Molecular weight markers and the protein names are indicated.

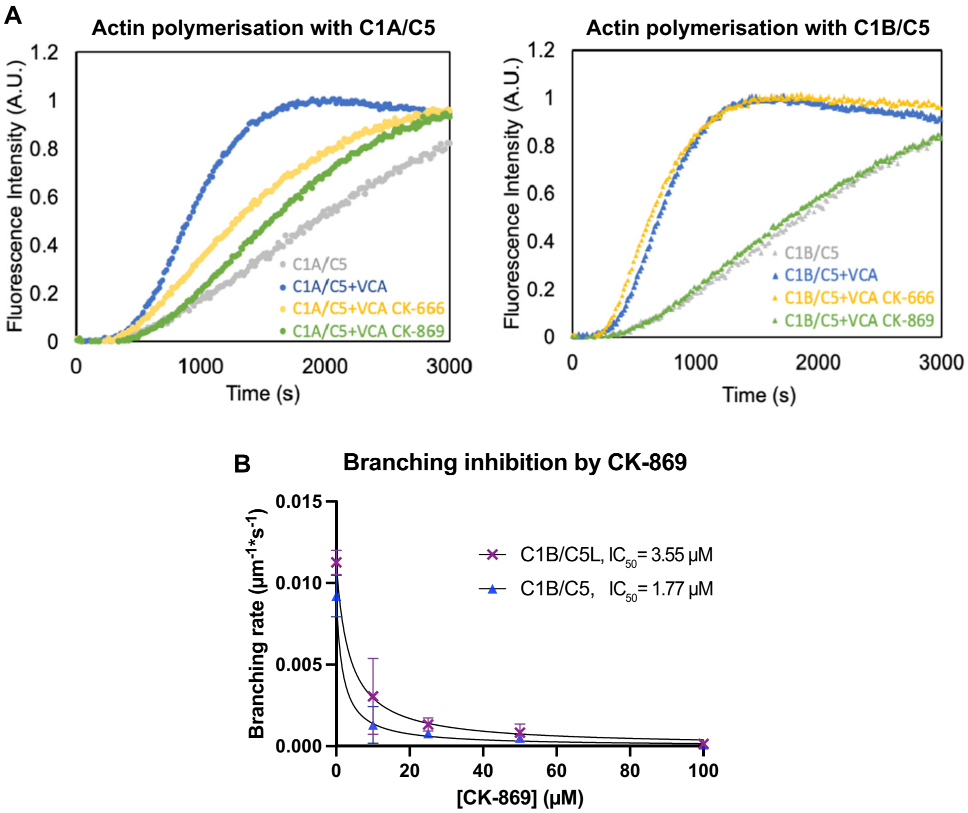

Figure EV3. Inhibition of actin branches by CK-666 and CK-869.

(**A**) Representative plots showing the polymerisation of pyrene actin (Fluorescence Intensity) when Arp2/3 complexes containing ArpC1A/C5 (left) or ArpC1B/C5 (right) are activated by GST-N-WASP in the absence (blue) or presence of 100 μM CK-666 (yellow) or CK-869 (green). (**B**) The branching rate of the Arp2/3 complex containing ArpC1B/C5 and ArpC1B/C5L (same as shown in Fig. 2C) were measured at the indicated CK-869 concentration. The data were fitted with equation Y = Bottom + (Top-Bottom)/(1 + (X/IC_{50})) to calculate the half-maximal inhibitory concentration (IC_{50}) of the CK-869. The points and error bars represent the mean and the standard deviation of at least two independent measurements.

 © The Author(s)

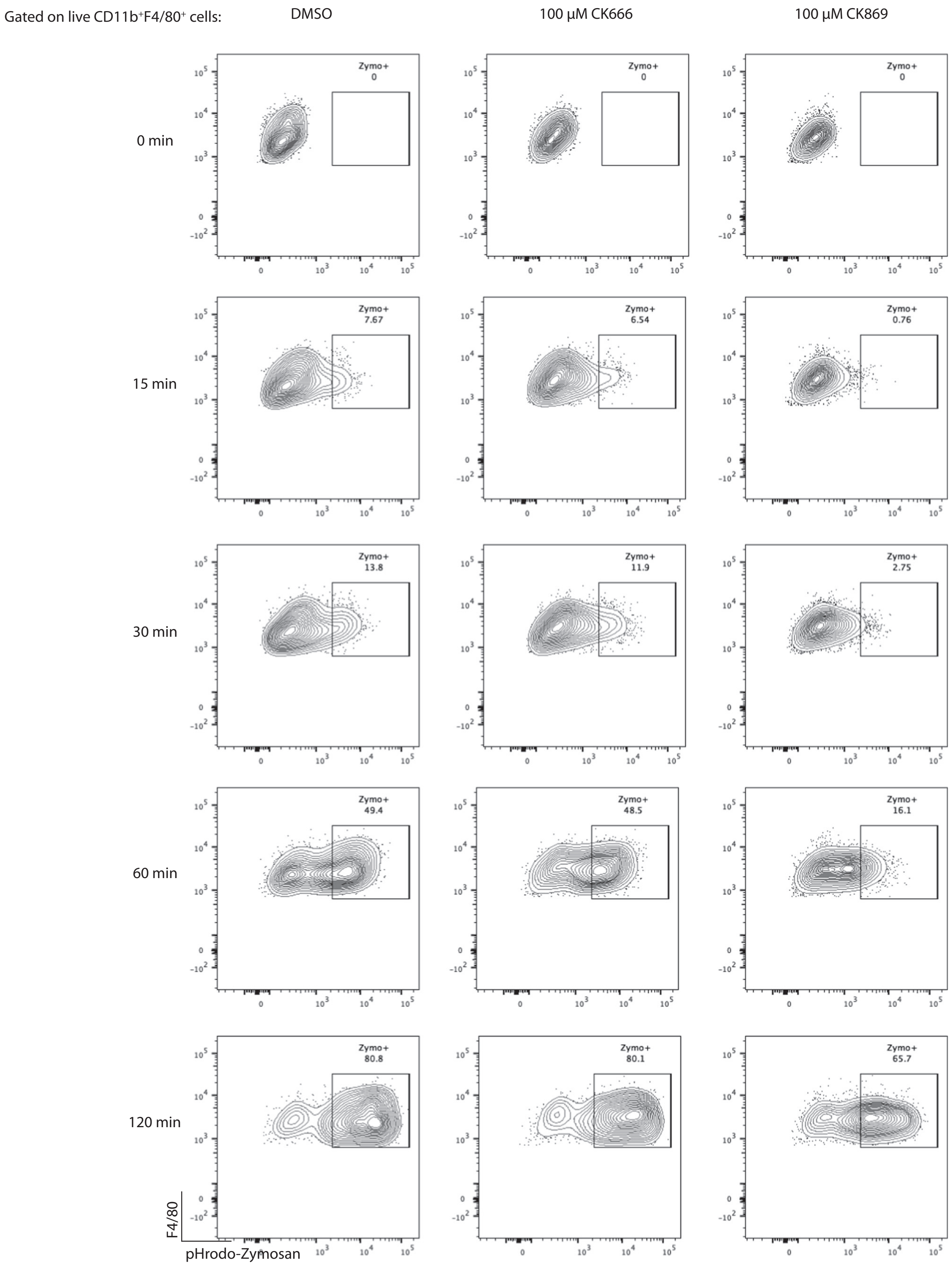

Figure EV4. Representative FACS plots of murine bone marrow-derived macrophage phagocytosis after treatment with DMSO (control) or 100 µM CK-666 or CK-869.

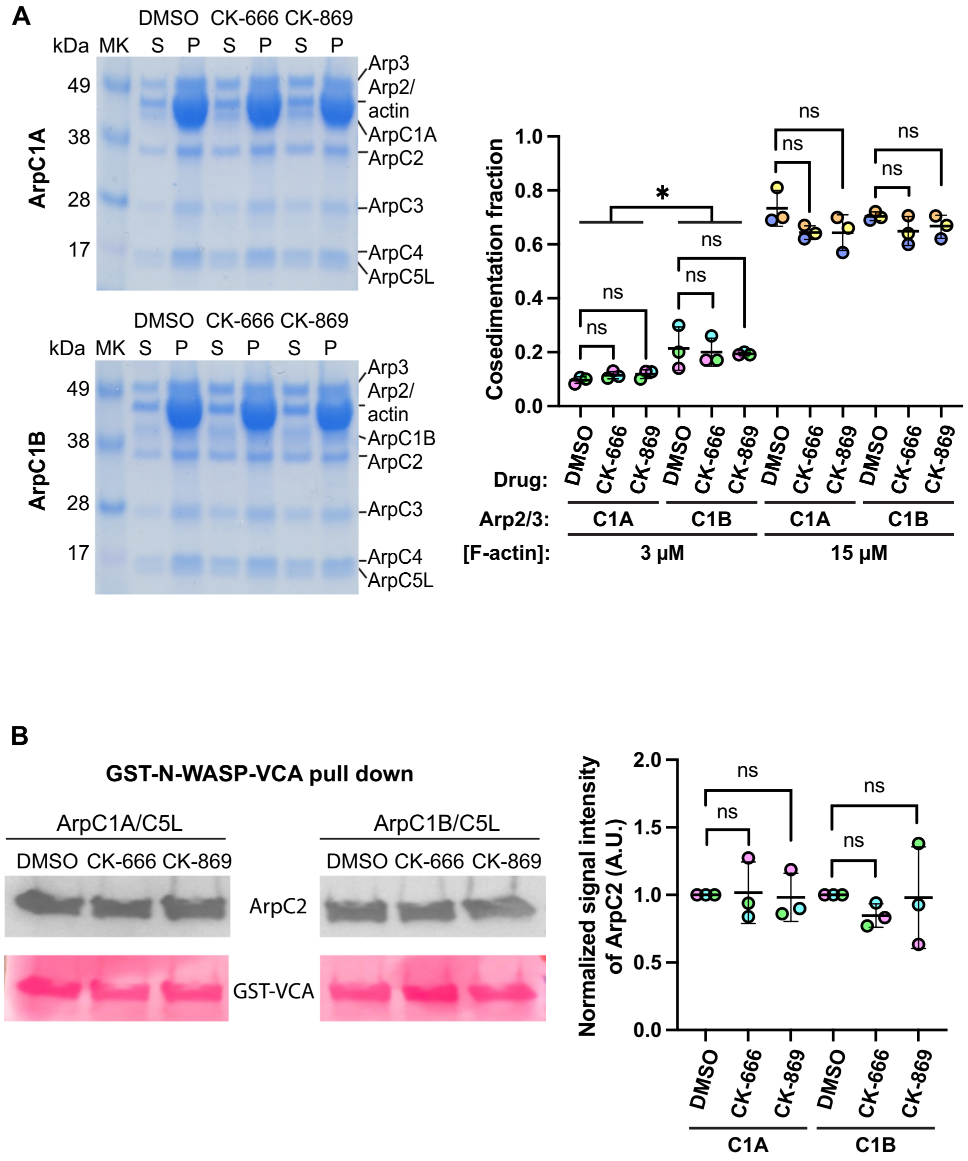

Figure EV5. Analysis of Arp2/3 complex binding to actin filaments or VCA.

(**A**) The left panel shows representative Coomassie-stained protein gels of the co-sedimentation of Arp2/3 complexes containing ArpC5L together with ArpC1A (top) or ArpC1B (bottom) with 15 µM actin (P pellet) in the absence (DMSO) or presence of 100 µM CK-666 or CK-869. The supernatant (S) contains unbound Arp2/3 complexes and non-pelleted G-actin. MK represents the molecular weight markers. The right graph shows the quantification of the mean co-sedimentation fraction of the indicated Arp2/3 complexes in the absence (DMSO) or presence of CK-666 or CK-869 from three independent experiments at 3 and 15 µM actin, with the error bars indicating the standard deviation. Two-tailed paired *t*-test was used to analyse the statistical significance. *p value < 0.05. ns not significant. (**B**) Immunoblots (left panels) using an ArpC2 antibody demonstrate that 100 µM CK-666 or CK-869 does not impact the interaction of Arp2/3 complexes ArpC5L together with ArpC1A (top) or ArpC1B (bottom) with GST-N-WASP-VCA (Ponceau - red). The right graph shows the quantification of the mean pull-down fraction of the indicated Arp2/3 complexes in the absence (DMSO) or presence of CK-666 or CK-869 from three independent experiments, with the error bars indicating the standard deviation. Two-tailed paired *t*-test was used to analyse the statistical significance. ns not significant.

© The Author(s)

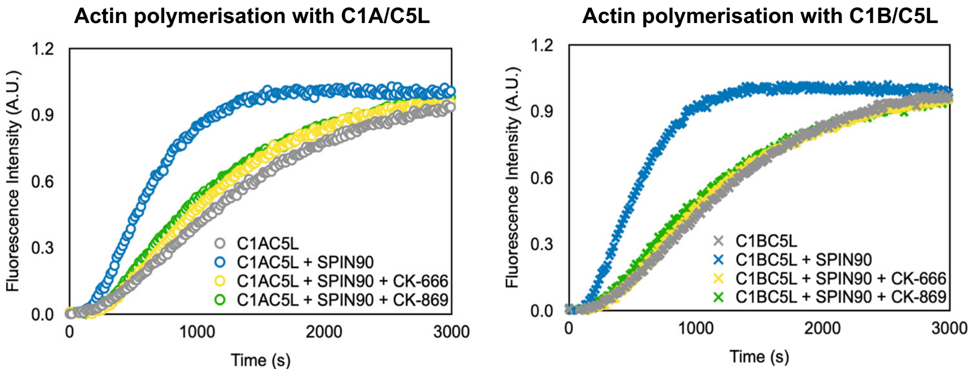

Figure EV6. Inhibition of SPIN90-Arp2/3 nucleated linear actin filaments.

Representative plots showing the polymerisation of pyrene actin (Fluorescence Intensity) when Arp2/3 complexes containing ArpC1A/C5L (left) or ArpC1B/C5L (right) are activated by SPIN90-Cter in the absence (blue) or presence of 100 µM CK-666 (yellow) or CK-869 (green).
